# Changes in Serum and Salivary Proteins in Canine Mammary Tumors

**DOI:** 10.3390/ani10040741

**Published:** 2020-04-24

**Authors:** Lorena Franco-Martínez, Andrea Gelemanović, Anita Horvatić, María Dolores Contreras-Aguilar, Roman Dąbrowski, Vladimir Mrljak, José Joaquín Cerón, Silvia Martínez-Subiela, Asta Tvarijonaviciute

**Affiliations:** 1 Interdisciplinary Laboratory of Clinical Pathology, Interlab-UMU, University of Murcia, 30100 Murcia, Spain; Lorena.franco2@um.es (L.F.-M.); mariadolores.contreras@um.es (M.D.C.-A.); jjceron@um.es (J.J.C.); asta@um.es (A.T.); 2 Mediterranean Institute for Life Sciences (MedILS), 21000 Split, Croatia; andrea.gelemanovic@gmail.com; 3 Faculty of Veterinary Medicine, University of Zagreb, Heinzelova 55, 10 000 Zagreb, Croatia; horvatic.ani@gmail.com (A.H.); vmrljak@vef.hr (V.M.); 4 Department and Clinic of Animal Reproduction, Faculty of Veterinary Medicine, University of Life Sciences in Lublin, 30 Gleboka St., 20-612 Lublin, Poland; roman.dabrowski@up.lublin.pl

**Keywords:** biomarker, cancer, canine mammary tumors, dog, proteomics, saliva, serum

## Abstract

**Simple Summary:**

The present study describes for the first time the differences in the serum and saliva proteomes between healthy bitches and bitches with mammary tumors using a high-throughput proteomic approach. More than 1000 proteins were identified and 35 in serum and 49 in saliva were significantly modulated. Additionally, their related pathways were discussed in order to improve understanding of the pathophysiology of the disease and one protein, serum albumin, was further validated. The results of the present study could be a source of potential biomarkers for canine mammary tumors in saliva and serum and increase the knowledge on the pathophysiology of the disease.

**Abstract:**

The aim of this study was to evaluate changes in serum and saliva proteomes in canine mammary tumors (CMT) using a high-throughput quantitative proteomic analysis in order to potentially discover possible biomarkers of this disease. Proteomes of paired serum and saliva samples from healthy controls (HC group, *n* = 5) and bitches with CMT (CMT group, *n* = 5) were analysed using a Tandem Mass Tags-based approach. Twenty-five dogs were used to validate serum albumin as a candidate biomarker in an independent sample set. The proteomic analysis quantified 379 and 730 proteins in serum and saliva, respectively. Of those, 35 proteins in serum and 49 in saliva were differentially represented. The verification of albumin in serum was in concordance with the proteomic data, showing lower levels in CMT when compared to the HC group. Some of the modulated proteins found in the present study such as haptoglobin or S100A4 have been related to CMT or human breast cancer previously, while others such as kallikrein-1 and immunoglobulin gamma-heavy chains A and D are described here for the first time. Our results indicate that saliva and serum proteomes can reflect physiopathological changes that occur in CMT in dogs and can be a potential source of biomarkers of the disease.

## 1. Introduction

Mammary tumors are the most common cancer in intact female dogs, accounting for almost 50% of all tumors [1]. Approximately half of all canine mammary tumors (CMT) are diagnosed as malignant by histological examination, leading to death due to distant metastases in many cases [2]. Several conditions such as hormonal imbalance, viruses, obesity, diet, genetic components, and oxidative stress contribute to CMT [1,3], although the pathogenesis has not been fully elucidated to date. It is known that many biological characteristics of mammary tumors are shared between humans and dogs such as histopathologic features, biological behaviour, metastatic pattern, antigenic phenotype and expression patterns of proliferating cell nuclear antigen, cytokeratins, and calponin [4]. Therefore, dogs have been proposed as an ideal model for the investigation of human breast cancer [5]. Furthermore, since humans and dogs share the same environment and are exposed to the same cancerogenic factors, spontaneously occurring malignancies in dogs have been considered as a valuable experimental model for investigation of human neoplasias [6].

Saliva is a non-invasive biological fluid that can reflect both systemic and local health-related conditions and possesses several benefits when used for sampling instead of invasive methods such as blood sampling. The advantages of using saliva include that it is a safer method for personnel and patients, it is easier to collect, and collecting saliva reduces sampling stress as it is pain-free [7]. In humans, proteomic analyses of saliva have proved to be a useful technique for discovering disease-related biomarkers in different pathologies including cancer [8]. In dogs, gel-free proteomics approaches such as Tandem Mass Tag (TMT) have been successfully used in serum and/or saliva in different infectious diseases such as pyometra [9], parvoviral enteritis [10] and leishmaniosis [11], as well as for metabolic diseases such as obesity [12].

Changes in serum and saliva proteomes in women with breast cancer have been described. In serum, higher levels of histone deacetylase proteins were detected in women with recurrent breast cancer in comparison to non-recurrent cases [13]. In saliva, Delmonico et al. [14] identified differences in alpha-2-macroglobulin, ceruplasmin, haptoglobin, hemopexin and vitamin D-binding, among others, in women with impalpable breast cancer in comparison to healthy women; and Giri et al. [15] identified 166 proteins with different expression in saliva of women with breast cancer in comparison to healthy controls [15]. In dogs, two studies were reported in which 2-DE gel proteomic analyses of canine mammary tumor tissues were performed. In one of the studies [16], of 21 proteins differentially modulated in healthy and diseased dogs, 19 were previously reported for human breast cancer. The second study [17] compared normal canine mammary gland tissue proteomes with tissues at different stages of malignant progression, identifying 48 proteins with significant changes. However, to the best of the authors’ knowledge, there are no studies addressing changes in serum or saliva proteomes in dogs with mammary tumors.

The current study aims to explore for the first time changes in paired serum and saliva proteomes in bitches with mammary tumors compared to healthy ones by using TMT label-based gel-free proteomic approach. This study is expected to increase the knowledge on the pathogenesis of CMT and potentially discovering possible biomarkers for the detection of this disease.

## 2. Materials and Methods

### 2.1. Animals

A total of 10 client-owned bitches presenting at private veterinary clinics in Murcia, Spain, during January 2019 were involved in this study. A group of five bitches (3 mixed breeds, 1 Pinscher and 1 German shepherd; all spayed; aged 9.7 ± 3.6 years old) classified as healthy based on a complete physical examination, haematology and biochemistry were included as the control group (HC). Five bitches (4 mixed breeds and 1 Pinscher; all spayed; aged 12.0 ± 1.93 years old) diagnosed with mammary tumors (3 simple tubulopapillary carcinomas and 2 simple tubular carcinoma) were included as the mammary tumor (CMT) group. All dogs were submitted to general physical examination and general haematology and biochemistry were performed. Tumor staging was based on the ‘Tumor, Node, Metastasis classification system’ (TNM) [18], and all cases were diagnosed as grades 1 and 2 (tumor diameter ≤3 cm, and absence of regional lymph node or distant metastases). Dogs were excluded if they were spayed less than one year ago, if there was possibility of concurrent diseases based on physical examination and laboratory analyses, or if gingivitis was detected. None of the serum and saliva samples showed haemolysis. All dogs with TNM were treated by surgical removal of the neoplasm and were alive and without relapse 6 months after the surgery.

All the procedures were approved by the ethics committees of the University of Murcia and the Ministry of Agriculture, Livestock, Fishing and Aquaculture of the Region of Murcia (A13170503).

### 2.2. Serum and Saliva Sampling

Surplus serum remainings after routine clinical biochemical analyses were stored at −80 °C until their use for the present study. Venipuncture of the jugular or cephalic vein was performed for blood collection. Whole blood was stored in tubes containing a coagulation activator and a gel separator and kept at room temperature (25 °C) until visible clot reaction. Samples were centrifuged (3500× *g*, 10 min) and the supernatants were stored at −80 °C until analysis.

Saliva samples were obtained as previously reported [19]. In brief, a small sponge was placed into the mouth and, when it was thoroughly moistened, it was then removed and placed into collection devices (Salivette saliva collection tube/V-Bottom, Sarstedt, Aktiengesellschaft & Co, Nümbrecht, Germany). After the samples were centrifuged (3000× *g*, 10 min, 4 °C), the supernatants were stored at −80 °C until analysis [20]. At least 0.2 mL of saliva was collected from each patient.

### 2.3. Proteomics Study of Saliva and Serum Samples and LC–MS/MS Analysis

A total of 35 µg of protein per sample was subjected to reduction, alkylation, digestion and labelling using 6-plex Tandem Mass Tag reagents, according to manufacturer instructions (Thermo Scientific) as described previously [10,11]. In brief, after protein determination using Bradford assay [21], 35 µg of sample and internal standards (consisting of a pool of 35 µg of each sample) were reduced for 1 h with 200 mM DTT (Sigma-Aldrich), alkylated for 30 min with 375 mM iodoacetamide (Sigma-Aldrich) and precipitated with ice-cold acetone (VWR, Llinars del Vallés, Barcelona, Spain). The next day, the samples were centrifuged, the acetone was decanted and the pellets were resuspended with 50 μL of 100 mM TEAB buffer and subsequently digested with trypsin (Promega, Madison, WI, USA) overnight at 37 °C (2.5 μg of trypsin per 100 μg of protein). The TMT labelling reagents were resuspended in LC–MS-grade anhydrous acetonitrile (Thermo Scientific, Waltham, MA, USA) and mixed with each sample for 1 h at room temperature. The reaction was quenched by adding 5% hydroxylamine (Thermo Scientific) for 15 min and the samples were combined at equal amounts. Then, 6 μg of each mixed sample set was placed in a well of a microplate, vacuum-dried and stored at −80 °C before further LC–MS/MS analysis. LC−MS/MS analysis was performed using the Dionex Ultimate 3000 RSLC nano-flow system (Dionex, Camberley, UK) and an Orbitrap Q Exactive Plus mass spectrometer (Thermo Fisher Scientific), as described elsewhere [22]. The SEQUEST algorithm, Proteome Discoverer (version 2.0., Thermo Fisher Scientific, Waltham, MA, USA), was used for peptide identification and relative quantification. A NCBI database search for *Canis Lupus* FASTA files was performed considering two missed trypsin cleavage sites, a precursor tolerance of 10 ppm and a fragment mass tolerance of 0.02 Da. The Percolator algorithm within the Proteome Discoverer workflow was used to determine the false discovery rate (FDR) for peptide identification, which was set at 1%.

### 2.4. Validation of Serum Biomarkers

For validation of serum biomarkers detected by proteomic analysis, serum samples from 25 bitches that were presented to the Department and Clinic of Animal Reproduction, University of Life Sciences, Lublin, Poland, were employed—healthy controls (*n* = 10; aged 7.5–11) and dogs with CMT stage 1–2 tumors (*n* = 15; aged 8–13).

Albumin in serum was selected for validation since it is commonly performed in routine biochemistry analysis in our laboratory and, therefore, that data were already available. Serum albumin was determined using a commercially available kit (Albumin OSR 6102; Olympus Life and Material Science Europe GmbH, Irish branch, Ennis, Ireland), according to manufacturer instructions.

### 2.5. Statistical Analysis

All statistics were performed using R v3.2.2 [23]. First, proteins with fewer than two unique peptides and proteins with >90% missing data were removed from the analysis. Sample outliers were detected for each of the proteins using Dixon’s test from R package *outliers* v0.14 [24]. If a sample outlier was significant (*p* < 0.05), it was removed from further analysis. As the majority of the analysed proteins did not follow normal distribution, as tested by the Shapiro–Wilk test, the Wilcoxon–Mann–Whitney test was performed in order to test for differences in protein abundance between groups. The fold change between the two groups was calculated as the mean protein abundance for the CMT group divided by the mean protein abundance for the HC group.

For the validation of serum biomarkers, the D’Agostino and Pearson omnibus normality test was employed to determine the distribution of data and, since data were not normally distributed, the non-parametric statistical Mann–Whitney U (two-way) test was used to compare between groups.

### 2.6. Bioinformatics Analysis

The proteins’ GI accession numbers were converted into official gene symbols either using the DAVID conversion tool (https://david.ncifcrf.gov/conversion.jsp), UniProtKB ID mapping (https://www.uniprot.org/uploadlists/) or the SEQUEST search engine implemented into Proteome Discoverer [25]. Genes encoding the differentially abundant proteins in the CMT and HC groups were used to determine the GO terms overrepresented in CMT using the Protein Analysis Through Evolutionary Relationships (PANTHER) classification tool (http://www.pantherdb.org/).

Heatmaps were designed using R package *pheatmap* v1.0.12 [26].

## 3. Results

### 3.1. Proteomic Analysis in Serum

**Figure A1:**
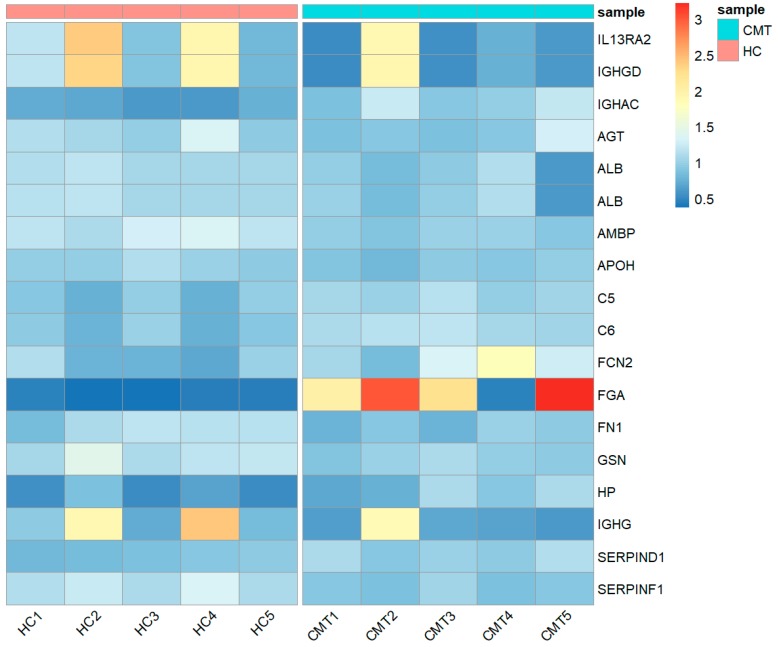
Heatmap showing the relative abundance (color) of serum proteins in healthy (HC) dogs and dogs with canine mammary tumors (CMT).

After the removal of proteins with fewer than 2 unique peptides, NMT <5% FDR, missing data and outliers, 379 serum proteins remained for statistical analysis (Table A1). The Wilcoxon–Mann–Whitney test revealed statistically significant different abundances in the HC and CMT groups for 35 proteins, corresponding to 16 unique genes after the removal of duplicates and isoforms, as summarized in Table 1 and Figure A1. The 35 proteins differentially expressed in serum in CMT and HC were used for subsequent bioinformatics analysis in terms of functional clusters, according to the PANTHER classification system (Table 2). The identified differentially modulated proteins in CMT and HC had three molecular functions: binding (40%), catalytic activity (30%) or molecular function regulators (30%). Three different biological processes were involved: 66% of proteins were involved in cellular process, 17% of proteins in biological adhesion and another 17% in localization. Regarding biological pathways, half of the proteins were involved in blood coagulation, while 25% were involved in the plasminogen activating cascade and in angiotensin II-stimulated signalling through G protein and beta-arrestin. Regarding protein class, 50% of proteins were enzyme modulators, followed by transfer/carrier proteins (17%), signalling molecules (17%), and receptors (16%).

Of the sixteen modulated proteins, seven were upregulated and nine were downregulated in CMT. Proteins with a fold increase greater than 2 were fibrinogen A-alpha chain (*FGA*) and IgA heavy chain constant region [*Canis lupus familiaris*]. The most downregulated proteins in CMT were interleukin-13 receptor subunit alpha-2 precursor [*Canis lupus familiaris*], immunoglobulin gamma-heavy chain A [*Canis lupus familiaris*], immunoglobulin gamma-heavy chain D [*Canis lupus familiaris*], and interleukin-13 receptor subunit alpha-2 precursor [*Canis lupus familiaris*].

### 3.2. Proteomic Analysis in Saliva

**Figure A2:**
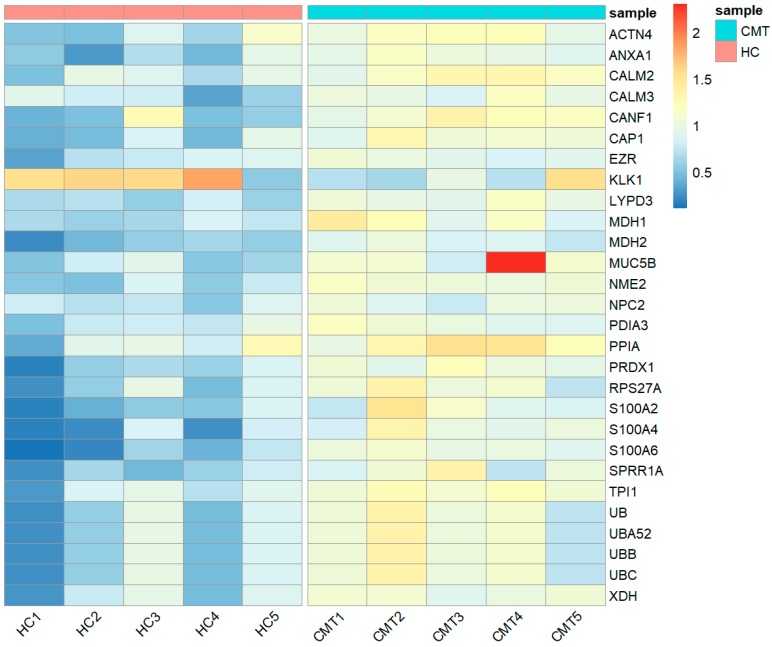
Heatmap showing the relative abundance (color) of salivary proteins in healthy (HC) dogs and dogs with canine mammary tumors (CMT).

After the removal of proteins with fewer than two unique peptides, NMT <5% FDR, missing data and outliers, 730 proteins remained for statistical analysis (Table A2). The Wilcoxon–Mann–Whitney test identified 49 proteins (corresponding to 28 unique genes) with different abundances between the HC and CMT groups (*p* < 0.05), as summarized after the removal of duplicates and isoforms in Table 3 and Figure A2. Proteins differentially expressed in saliva between CMT and HC were used for subsequent bioinformatics analysis in terms of functional clusters, according to the PANTHER classification system, as shown in Table 2. The identified differentially modulated proteins in CMT and HC had five molecular functions: binding (37%), catalytic activity (28%), structural molecule activity (21%), molecular function regulators (7%), and molecular transducer activity (7%). Furthermore, proteins were involved in nine different biological processes—with 35%, 22%, and 13% of proteins involved with cellular process, metabolic process, and localization, respectively. Most proteins were involved in nucleic acid binding (50%), although there were also cytoskeletal proteins, isomerases and transfer/carrier proteins. Regarding biological pathways, the differentially modulated proteins were involved in *de novo* purine biosynthesis, de novo pyrimidine deoxyribonucleotide biosynthesis, *de novo* pyrimidine ribonucleotide biosynthesis, glycolysis (P00024), and salvage pyrimidine ribonucleotides (20% of proteins for each).

Of the 28 proteins, 27 were upregulated and 1 was downregulated. Protein S100-A6 (*S100A6*), major allergen Can f1 (*CANF1*), protein S100-A4 (*S100A4*), malate dehydrogenase (*MDH2*), and protein S100-A2 (*S100A2*) were the most upregulated proteins in MT, with >2 fold change. Kallikrein-1 (*KLK1*) was the only protein that was downregulated in the CMT group.

Finally, when proteomic results were compared between saliva and serum, the abundance of no protein changed statistically significantly in both serum and saliva.

### 3.3. Validation of Albumin

When the CMT and HC groups were compared, lower circulating concentrations of albumin were found in bitches with CMT (median (25–75th percentile), 2.9 (2.6–3.2) g/dL) in comparison to healthy controls (3.1 (3.1–3.4) g/dL) (*p* = 0.0173).

## 4. Discussion

In this report, serum and salivary proteomes showed changes in bitches with CMT when compared to healthy individuals. Some of the proteins that were modulated in the present study such as S100A4 or haptoglobin were also described in previous studies on canine mammary tumors [27] or human breast cancer [14,15,28,29], while other proteins including immunoglobulin gamma-heavy chains A and D or kallikrein-1 were described here for the first time as CMT-related proteins.

In serum, 35 of 379 proteins were differentially modulated in CMT. Fibrinogen A-alpha chain (*FGA*) was the most upregulated protein in serum in CMT. This protein is a degradation product of the alpha chain of fibrinogen [30] and its protective role against tumor growth and metastasis has been proposed previously [31]. Increases in fibrinogen degradation have been described in neoplasms and seem related to an increase in fibrin deposition in the tumor [32]. *FGA* has been proposed as a biomarker for HER2-positive breast cancer [33], although it can also appear increased in oral cancer [34] and in infectious diseases such as dirofilariosis or leishmaniosis [35].

The two most downregulated proteins in serum of bitches with CMT were interleukin-13 receptor subunit alpha-2 precursor and immunoglobulin gamma-heavy chains A and D. Interleukins are key immunoregulatory and anti-inflammatory cytokines in tumor cells, produced by different immune cells such as B, T, mast cells, dendritic or natural killer cells [36]. In dogs, the upregulation of interleukin-13 receptor subunit alpha-2 genes was observed in macrophages grown as a co-culture with canine mammary cancer cells [37]. In human breast cancer tissues, overexpression of interleukin-13 receptor subunit alpha-2 precursor has been suggested as an independent predictor of poorer outcome, playing important roles in cancer cell survival and progression, although it was dependent on the breast cancer subtype [38]. In the present study, dogs had cancers of low TNM grade and did not have relapses for at least 6 months, which could explain at least partially the observed low levels of this protein. Immunoglobulin gamma is the most abundant class of antibodies, predominant in the immune secondary response and participating in different important roles such as the activation of the complement cascade, or mediation of antibody-dependent cell cytotoxicity [39]. There is a lack of information on the possible relation between IgG and CMT and, therefore, further studies would be diserable in order to discern the possible reasons for the IgG downregulation observed in our study.

Additionally in serum, modulations of known acute-phase proteins (APP) in dogs with increases in haptoglobin and decreases in albumin were observed in the proteomic analysis of this study. While haptoglobin is considered a moderate APP in dogs that increases by approximately 2- to 5-fold, albumin is a negative APP that decreases during inflammation [40]. In agreement with our results, increases in serum haptoglobin concentrations were observed in dogs with mammary tumors when compared to healthy controls [41,42], as well as in dogs with hemolymphatic, mesenchymal, and epithelial tumors [40,43,44]. The downregulation of albumin in serum in CMT bitches was further verified previously by a commercially available kit using an automated analyser, showing a decrease in albumin in dogs with CMT in comparison to controls.

In saliva, three proteins of the S100A family, namely S100A2, S100A4, and S100A6, were among the most upregulated proteins in bitches with mammary tumors when compared to healthy controls. S100A proteins are a family of calcium-binding proteins with potential roles in different cancers including breast [45]. In breast cancer, the positive correlation between S100A4 expression and cancer progression has been described in several studies [28,46,47]. A controversial role in carcinogenesis has been described for S100A2, which has been proposed both as a tumor suppressor [48] and a tumor promotor [49]. Higher tissue expression of S100A2 was significantly correlated with worse overall survival in ovarian cancer patients [15], and S100A2 protein expression in saliva was observed to be higher in oral squamous cell carcinoma patients in comparison to other oral potentially malignant disorders. S100A6 expression has been associated with worse prognosis or the progression of several cancers including oncogynecology [50], colorectal [51], gastric [52], and renal [53]. In contrast, a recent study suggests a relation between S100A6 expression and better prognosis in breast cancer [54]. However, to the best of our knowledge, this is the first report in which the S100A family is described in saliva of bitches with mammary tumors. Based on our results and previous studies in women, evaluation of the S100A family as possible biomarkers of prognosis and evolution of mammary tumors in dogs could be of interest in order to evaluate whether dogs with poorer prognosis can have higher values of these proteins.

Calmodulin-like proteins, namely CALM2 and CALM3, were also upregulated in saliva of bitches with mammary tumors. These proteins are described here as modulated in saliva in CMT for the first time. Previous studies in humans reported overexpression of calmodulins in serum of patients with breast cancer [55] and the use of calmodulin antagonists as a therapeutic strategy [56].

Kallikrein-1-like (KLK1) protein was the only downregulated protein in saliva of bitches with CMT. Both kallikrein genes and proteins might promote or inhibit cancer cell growth, angiogenesis, invasion and metastasis by different mechanisms [57]. Therefore, the role of KLK in cancer is still controversial, with several studies associating KLK with better or worse income. For example, the *in vitro* inhibition of KLK1 suppresses the invasiveness of breast cancer cells, and therefore it could be a defence mechanism [58]. On the other hand, KLK-mediated degradation of extracellular matrix proteins facilitates tumor cell invasion and metastasis [59], and higher serum levels of other kallikreins such as KLK5 [60], KLK10 [61], and KLK14 [62] have been found in women with breast cancer when compared to healthy ones. Hormonal regulation of KLK1 expression was suggested in human prostate and breast tissues [63] and its salivary secretion was proposed as modulated by age [64]. Nevertheless, to the best of the author’s knowledge, this is the first report describing a downregulation of KLK1 in saliva in CMT, and the possible causes for its modulation should be further studied.

When serum and salivary proteomes were compared, approximately twice as many proteins were identified in saliva compared to serum (730 in saliva vs. 379 in serum). This is in agreement with previous studies reporting a higher number of proteins in saliva [65], and indicates that saliva can provide opportunities for the identification of new non-invasive biomarkers. This fact also suggests that a high number of proteins that appear in saliva are not transported from blood but may come from salivary glands, nasal and bronchial secretions, gingival crevicular fluid among others, and, thus, their origin should be investigated in future studies. In our study, none of the proteins were significantly differentially modulated in both serum and saliva at the same time, which reinforces the possible different origin of many proteins in serum and saliva.

The results of the present study suffer from some limitations. First, the included animals were client-owned bitches of different breeds, which may have influenced our results and increased variability since, for example, higher expression of p53 (which is generally associated with poorer overall survival) has been reported in large-breed dogs [66]. In addition, although these biomarkers were not studied in our reports, cancer-related biomarkers including estrogen receptor alpha (ERα), progesterone receptor (PR) and human epidermal growth factor receptor 2 (HER2) differ between the type and subtype of CMT and could have a potential influence in the results [67,68]. Finally, the sample size was relatively small, although it was greater than the minimum of three biological replicates recommended for proteomic studies [69]. Therefore, this study should be considered as a pilot study, and further research with larger populations are needed in order to confirm our findings and to evaluate the potential influence of variables such as age, breed, tumor type based on immunophenotype, and hormonal influence in addition to other possible confounding factors such as other types of neoplasms, different diseases or the presence of oral alterations such as gingivitis.

## 5. Conclusions

Dogs with CMT have changes in serum and saliva proteomes as compared to healthy dogs. Bioinformatic analyses in serum and saliva showed that most modulated proteins have binding or catalytic activity molecular functions. Proteins such as kallikrein-1 and immunoglobulin gamma-heavy chains A and D were related to CMT in this study for the first time, and could be considered as potential novel CMT biomarkers. In addition, some of the modulated proteins found in the present study such as haptoglobin or S100A4 have been related to CMT or human breast cancer previously, which confirms the validity of our study design for CMT evaluation and the use of CMT as a model for breast cancer research. Overall, data presented in this report reflect the changes that occur in the proteome of serum and saliva in dogs with CMT and could be a potential source for the study of new biomarkers for this disease.

## Figures and Tables

**Table 1:** Proteins downregulated and upregulated in serum. In serum, 35 proteins were initially identified with statistically significant different abundances in CMT and HC. After the removing of duplicates and isoforms, there were 9 downregulated and 7 upregulated proteins.

GI Accession	Gene Symbol	Description	*p*-Value	Fold Change	Mean (SD)	Mean (SD)
					HC	CMT
**Proteins upregulated in CMT when compared to HC**						
345791439	*SERPIND1*	Heparin cofactor 2	0.032	1.168	0.874 (0.061)	1.021 (0.092)
1239925760	*C5*	Complement component C5	0.016	1.194	0.875 (0.112)	1.045 (0.066)
1239899336	*C6*	Complement component C6	0.008	1.254	0.883 (0.109)	1.107 (0.068)
73967778	*FCN2*	Ficolin-2	0.048	1.443	0.873 (0.177)	1.260 (0.366)
258499	*HP*	Haptoglobin heavy chain	0.032	1.458	0.620 (0.151)	0.904 (0.182)
60734606	*IGHAC*	Immunoglobulin alpha heavy chain constant region	0.008	2.030	0.501 (0.077)	1.017 (0.223)
1304047	*FGA*	Fibrinogen A-alpha chain	0.016	5.683	0.394 (0.060)	2.239 (1.186)
**Proteins downregulated in CMT when compared to HC**						
16607715	*IL13RA2*	Interleukin-13 receptor subunit alpha-2	0.016	0.42	1.434 (0.686)	0.603 (0.102)
16607648	*IGHG*	Immunoglobulin gamma-heavy chain D	0.016	0.42	1.423 (0.672)	0.602 (0.099)
119637837	*SERPINF1*	Pigment epithelium-derived factor	0.008	0.78	1.178 (0.106)	0.915 (0.068)
1272414	*FN1*	Fibronectin	0.016	0.78	1.143 (0.041)	0.888 (0.103)
345777712	*AMBP*	Alpha-1-microglobulin/bikunin precursor	0.008	0.78	1.219 (0.100)	0.954 (0.051)
5821257	*AGT*	Angiotensinogen	0.016	0.81	1.094 (0.155)	0.886 (0.034)
3319897	*ALB*	Albumin	0.048	0.82	1.101 (0.059)	0.904 (0.193)
1239925762	*GSN*	Gelsolin	0.032	0.82	1.194 (0.141)	0.981 (0.074)
296089	*APOH*	Apolipoprotein H	0.032	0.90	1.009 (0.07)	0.911 (0.065)

Color represent relative abundance of the protein (red for downregulated, green for upregulated).

**Table 2:** Molecular function, biological process, pathways, and protein class as the number of genes and gene percentage of the differentially expressed proteins in the HC and CMT groups in serum and saliva based on the Protein Analysis Through Evolutionary Relationships (PANTHER) classification system.

Serum			Saliva		
	Gene	% Gene		Gene	% Gene
**Molecular Function**					
Binding (GO:0005488)	5	33.3	Binding (GO:0005488)	10	43.5
Catalytic activity (GO:0003824)	5	33.3	Catalytic activity (GO:0003824)	9	39.1
Molecular function regulator (GO:0098772)	4	26.7	Molecular function regulator (GO:0098772)	2	8.7
Molecular transducer activity (GO:0060089)	1	6.7	Structural molecule activity (GO:0005198)	2	8.7
**Biological Process**					
Biological regulation (GO:0065007)	4	28.6	Biological adhesion (GO:0022610)	2	4.2
Cellular process (GO:0009987)	4	28.6	Biological regulation (GO:0065007)	4	8.3
Localization (GO:0051179)	1	7.1	Cell population proliferation (GO:0008283)	1	2.1
Metabolic process (GO:0008152)	4	28.6	Cellular component organization or biogenesis (GO:0071840)	3	6.3
Response to stimulus (GO:0050896)	1	7.1	Cellular process (GO:0009987)	12	25
			Developmental process (GO:0032502)	2	4.2
			Growth (GO:0040007)	1	2.1
			Immune system process (GO:0002376)	2	4.2
			Localization (GO:0051179)	2	4.2
			Locomotion (GO:0040011)	1	2.1
			Metabolic process (GO:0008152)	9	18.8
			Multicellular organismal process (GO:0032501)	1	2.1
			Reproduction (GO:0000003)	1	2.1
			Reproductive process (GO:0022414)	1	2.1
			Response to stimulus (GO:0050896)	4	8.3
			Signaling (GO:0023052)	2	4.2
**Pathways**					
Angiotensin II-stimulated signaling through G proteins and beta-arrestin (P05911)	1	25	Adenine and hypoxanthine salvage pathway (P02723)	1	6.7
Blood coagulation (P00011)	1	25	B cell activation (P00010)	1	6.7
FAS signaling pathway (P00020)	1	25	CCKR signaling map (P06959)	1	6.7
Interleukin signaling pathway (P00036)	1	25	De novo purine biosynthesis (P02738)	1	6.7
			De novo pyrimidine deoxyribonucleotide biosynthesis (P02739)	1	6.7
			De novo pyrimidine ribonucleotides biosythesis (P02740)	1	6.7
			Glycolysis (P00024)	1	6.7
			Heterotrimeric G protein signaling pathway-Gi alpha and Gs alpha-mediated pathway (P00026)	1	6.7
			Heterotrimeric G protein signaling pathway-rod outer segment phototransduction (P00028)	1	6.7
			Integrin signalling pathway (P00034)	1	6.7
			Purine metabolism (P02769)	1	6.7
			Pyruvate metabolism (P02772)	1	6.7
			Salvage pyrimidine ribonucleotides (P02775)	1	6.7
			T cell activation (P00053)	1	6.7
			TCA cycle (P00051)	1	6.7
**Protein Class**					
Cytoskeletal protein (PC00085)	1	12.5	Calcium-binding protein (PC00060)	5	33.3
Intercellular signal molecule (PC00207)	1	12.5	Cytoskeletal protein (PC00085)	2	13.3
Protein-modifying enzyme (PC00260)	1	12.5	Extracellular matrix protein (PC00102)	1	6.7
Protein-binding activity modulator (PC00095)	4	12.550	Metabolite interconversion enzyme (PC00262)	5	33.3
Transfer/carrier protein (PC00219)	1	12.5	Protein-modifying enzyme (PC00260)	1	6.7
			Transmembrane signal receptor (PC00197)	1	6.7

**Table 3:** Proteins downregulated and upregulated in saliva. In saliva, 49 proteins were initially identified with statistically significant different abundances in CMT and HC. After the removing of duplicates and isoforms, there were 27 upregulated and 1 downregulated proteins.

GI Accession	Gene Symbol	Description	*p*-Value	Fold Change	Mean (SD)	Mean (SD)
					HC	CMT
**Proteins upregulated in CMT when compared to HC**						
356582247	*NPC2*	NPC intracellular cholesterol transporter 2 precursor	0.032	1.282	0.738 (0.129)	0.946 (0.108)
1418259105	*PDIA3*	Protein disulfide-isomerase A3	0.032	1.324	0.753 (0.170)	0.997 (0.115)
558757359	*EZR*	Ezrin	0.032	1.331	0.713 (0.222)	0.949 (0.086)
1418304395	*CALM3*	Calmodulin 3	0.016	1.445	0.690 (0.230)	0.997 (0.121)
1418197013	*CALM2*	Calmodulin 2	0.032	1.48	0.791 (0.208)	1.171 (0.158)
1418206782	*MUC5B*	Mucin 5B	0.040	1.498	0.673 (0.179)	1.008 (0.145)
73948247	*LYPD3*	Ly6/PLAUR domain-containing protein 3	0.008	1.51	0.673 (0.097)	1.016 (0.090)
76363530	*TPI1*	Triosephosphate isomerase	0.008	1.512	0.746 (0.266)	1.128 (0.091)
73947736	*ACTN4*	Alpha actinin 4	0.032	1.521	0.728 (0.273)	1.107 (0.121)
1418202786	*PPIA*	Peptidyl-prolyl cis-trans isomerase A	0.032	1.524	0.862 (0.322)	1.314 (0.240)
73980076	*XDH*	Xanthine dehydrogenase/oxidase	0.016	1.526	0.671 (0.288)	1.024 (0.076)
57092971	*MDH1*	Malate dehydrogenase, cytoplasmic	0.016	1.575	0.703 (0.100)	1.107 (0.239)
545510196	*NME2*	Nucleoside diphosphate kinase B	0.008	1.649	0.641 (0.165)	1.057 (0.053)
356582340	*RPS27A*	Ubiquitin-40S ribosomal protein S27a	0.032	1.665	0.626 (0.292)	1.042 (0.222)
5441519	*UBA52*	Ubiquitin-ribosomal protein L40 fusion protein	0.032	1.665	0.626 (0.292)	1.042 (0.222)
1239902229	*UBB*	Polyubiquitin-B	0.032	1.665	0.626 (0.292)	1.042 (0.222)
73995130	*UBC*	Polyubiquitin-C	0.032	1.665	0.626 (0.292)	1.042 (0.222)
558695394	*ANXA1*	Annexin A1	0.016	1.687	0.591 (0.250)	0.997 (0.092)
1239889921	*UB*	Ubiquitin	0.032	1.704	0.618 (0.259)	1.053 (0.215)
1418293878	*CAP1*	Adenylyl cyclase-associated protein 1	0.016	1.735	0.618 (0.251)	1.072 (0.132)
1418294943	*PRDX1*	Peroxiredoxin 1	0.008	1.783	0.580 (0.247)	1.034 (0.110)
1418213540	*SPRR1A*	Cornifin A	0.016	1.853	0.543 (0.214)	1.006 (0.235)
1418313584	*S100A2*	Protein S100-A2	0.032	2.008	0.514 (0.249)	1.032 (0.303)
1239955263	*MDH2*	Malate dehydrogenase, mitochondrial	0.008	2.052	0.481 (0.179)	0.987 (0.229)
1391723726	*S100A4*	Protein S100-A4	0.032	2.177	0.468 (0.337)	1.019 (0.191)
3121745	*CANF1*	Major allergen Can f 1	0.016	2.315	0.496 (0.067)	1.148 (0.150)
1239912330	*S100A6*	Protein S100-A6	0.008	2.356	0.424 (0.273)	0.999 (0.071)
**Proteins downregulated in CMT when compared to HC**						
1418303522	*KLK1*	Kallikrein-1-like	0.016	0.556	1.652 (0.126)	0.919 (0.379)

Color represent relative abundance of the protein (red for downregulated, green for upregulated).

## Appendix A

**Table A1:** Serum proteins identified in dogs with canine mammary tumors (CMT) and healthy controls (HC).

Gene Name	Accession Number	No. Unique Peptides	Protein Description	Fold Change (log2)	*p*-Value
*C6*	1418330227	7	complement component C6 [*Canis lupus dingo*]	0.326	0.008
*C6*	1239899336	7	complement component C6 [*Canis lupus familiaris*]	0.326	0.008
*IGHAC*	598107	9	IgA heavy chain constant region, partial [*Canis lupus familiaris*]	0.808	0.008
*SERPINF1*	119637837	5	pigment epithelium-derived factor [*Canis lupus familiaris*]	−0.364	0.008
*SERPINF1*	545510243	5	pigment epithelium-derived factor isoform X1 [*Canis lupus familiaris*]	−0.364	0.008
*AMBP*	1418322170	2	protein AMBP [*Canis lupus dingo*]	−0.354	0.008
*AMBP*	345777712	2	protein AMBP [*Canis lupus familiaris*]	−0.354	0.008
*N/A*	60734606	2	unnamed protein product, partial [*Canis lupus familiaris*]	1.021	0.008
*N/A*	60734607	5	unnamed protein product, partial [*Canis lupus familiaris*]	0.630	0.008
*AGT*	545494757	11	angiotensinogen [*Canis lupus familiaris*]	−0.190	0.016
*AGT*	5821257	3	angiotensinogen, partial [*Canis lupus familiaris*]	−0.304	0.016
*C5*	1418321303	31	complement C5 [*Canis lupus dingo*]	0.251	0.016
*C5*	1239925760	29	complement C5 [*Canis lupus familiaris*]	0.256	0.016
*FGA*	1304047	12	fibrinogen A-alpha chain, partial [*Canis lupus familiaris*]	2.507	0.016
*FGA*	1418336346	24	fibrinogen-alpha chain [*Canis lupus dingo*]	2.378	0.016
*FGA*	73978329	24	fibrinogen-alpha chain [*Canis lupus familiaris*]	2.378	0.016
*FN1*	1272414	11	fibronectin, partial [*Canis lupus familiaris*]	−0.364	0.016
*GSN*	1418322411	15	gelsolin isoform X1 [*Canis lupus dingo*]	−0.281	0.016
*GSN*	1418322413	15	gelsolin isoform X2 [*Canis lupus dingo*]	−0.281	0.016
*GSN*	1418322415	15	gelsolin isoform X3 [*Canis lupus dingo*]	−0.281	0.016
*IGHGD*	17066530	8	immunoglobulin gamma-heavy chain D [*Canis lupus familiaris*]	−1.073	0.016
*N/A*	16607648	6	unnamed protein product [*Canis lupus familiaris*]	−1.241	0.016
*IL13RA2*	16607715	6	interleukin-13 receptor subunit alpha-2 precursor [*Canis lupus familiaris*]	−1.250	0.016
*N/A*	16607724	8	unnamed protein product [*Canis lupus familiaris*]	−1.073	0.016
*N/A*	16607681	8	unnamed protein product, partial [*Canis lupus familiaris*]	−1.073	0.016
*APOH*	296089	13	apolipoprotein H; beta-2-glycoprotein I [*Canis lupus familiaris*]	−0.147	0.032
*GSN*	1239925762	14	gelsolin [*Canis lupus familiaris*]	−0.283	0.032
*HP*	258499	25	haptoglobin heavy chain, HpH chain [dogs, Peptide, 245 aa]	0.544	0.032
*SERPIND1*	345791439	11	heparin cofactor 2 [*Canis lupus familiaris*]	0.224	0.032
*SERPIND1*	1418264107	10	LOW QUALITY PROTEIN: heparin cofactor 2 [*Canis lupus dingo*]	0.224	0.032
*N/A*	16607678	3	unnamed protein product, partial [*Canis lupus familiaris*]	−1.184	0.032
*ALB*	3319897	2	albumin [*Canis lupus familiaris*]	−0.284	0.048
*FCN2*	73967778	4	ficolin-2 isoform X1 [*Canis lupus familiaris*]	0.529	0.048
*FCN2*	1239920689	4	ficolin-2 isoform X2 [*Canis lupus familiaris*]	0.529	0.048
*ALB*	1418328547	4	LOW QUALITY PROTEIN: serum albumin-like [*Canis lupus dingo*]	−0.276	0.048
*ALB*	2147092	2	albumin—dog (fragment)	−0.338	0.056
*FGB*	1418336465	24	fibrinogen beta chain [*Canis lupus dingo*]	1.936	0.056
*FN1*	1272412	8	fibronectin, partial [*Canis lupus familiaris*]	−0.290	0.056
*HP*	73957095	30	haptoglobin-like [*Canis lupus familiaris*]	0.493	0.056
*AOC3*	57091057	2	membrane primary amine oxidase isoform X1 [*Canis lupus familiaris*]	0.342	0.056
*AOC3*	1418340066	2	membrane primary amine oxidase isoform X2 [*Canis lupus dingo*]	0.342	0.056
*AOC3*	1239918266	2	membrane primary amine oxidase isoform X3 [*Canis lupus familiaris*]	0.342	0.056
*AOC3*	1239918269	2	membrane primary amine oxidase isoform X4 [*Canis lupus familiaris*]	0.342	0.056
*KLKB1*	1418255638	7	plasma kallikrein isoform X1 [*Canis lupus dingo*]	−0.195	0.056
*KLKB1*	1239938505	6	plasma kallikrein isoform X2 [*Canis lupus familiaris*]	−0.195	0.056
*HP*	123511	30	Haptoglobin beta chain	0.493	0.056
*RBP4*	928175781	6	retinol-binding protein 4 [*Canis lupus familiaris*]	−0.397	0.056
*F9*	163948	3	factor IX [*Canis lupus familiaris*]	0.276	0.057
*FGG*	545524897	19	fibrinogen gamma chain isoform X1 [*Canis lupus familiaris*]	2.093	0.095
*FGG*	73977992	19	fibrinogen gamma chain isoform X2 [*Canis lupus familiaris*]	2.093	0.095
*IGL-1*	1239964876	2	immunoglobulin lambda-1 light chain isoform X32 [*Canis lupus familiaris*]	−0.364	0.095
*IGL-1*	1239964884	2	immunoglobulin lambda-1 light chain isoform X37 [*Canis lupus familiaris*]	−0.364	0.095
*IGL-1*	1239964904	2	immunoglobulin lambda-1 light chain isoform X48 [*Canis lupus familiaris*]	−0.364	0.095
*IGL-1*	1239964906	2	immunoglobulin lambda-1 light chain isoform X49 [*Canis lupus familiaris*]	−0.364	0.095
*CPB2*	1418275904	2	carboxypeptidase B2 [*Canis lupus dingo*]	0.672	0.100
*C8A*	1418311120	3	complement component C8 alpha chain isoform X1 [*Canis lupus dingo*]	−0.394	0.100
*C8A*	359319377	3	complement component C8 alpha chain isoform X1 [*Canis lupus familiaris*]	−0.394	0.100
*C8A*	1418311122	3	complement component C8 alpha chain isoform X2 [*Canis lupus dingo*]	−0.394	0.100
*C8A*	1239902894	3	complement component C8 alpha chain isoform X2 [*Canis lupus familiaris*]	−0.394	0.100
*ACTC1*	57108093	3	actin, alpha cardiac muscle 1 [*Canis lupus familiaris*]	0.128	0.111
*ACTA1*	1418509163	3	actin, alpha skeletal muscle [*Canis lupus dingo*]	0.128	0.111
*ACTA1*	545494777	3	actin, alpha skeletal muscle [*Canis lupus familiaris*]	0.128	0.111
*ACTA2*	1418341514	3	actin, aortic smooth muscle [*Canis lupus dingo*]	0.128	0.111
*ACTB*	924183513	4	actin, cytoplasmic 1 [*Canis lupus familiaris*]	0.128	0.111
*ACTB*	545500782	4	actin, cytoplasmic 1 isoform X1 [*Canis lupus familiaris*]	0.128	0.111
*ACTB*	1418248118	4	actin, cytoplasmic 2 [*Canis lupus dingo*]	0.128	0.111
*ACTG1*	924442847	4	actin, cytoplasmic 2 [*Canis lupus familiaris*]	0.128	0.111
*ACTG2*	1418210682	3	actin, gamma-enteric smooth muscle [*Canis lupus dingo*]	0.128	0.111
*ACTB*	5597005	3	beta-actin [*Canis lupus familiaris*]	0.128	0.111
*C3*	928164703	4	complement C3 [*Canis lupus familiaris*]	−0.374	0.111
*LOC112676346*	1418260262	2	uncharacterized protein LOC112676346 [*Canis lupus dingo*]	−0.698	0.111
*F5*	1418316947	2	coagulation factor V [*Canis lupus dingo*]	0.299	0.114
*F5*	545504920	2	coagulation factor V [*Canis lupus familiaris*]	0.299	0.114
*C1qB*	1418507477	5	complement C1q subcomponent subunit B [*Canis lupus dingo*]	0.101	0.135
*ADIPOQ*	1418515478	3	adiponectin isoform X1 [*Canis lupus dingo*]	−0.302	0.151
*ADIPOQ*	1239977620	3	adiponectin isoform X2 [*Canis lupus familiaris*]	−0.302	0.151
*ADIPOQ*	54792748	3	adiponectin precursor [*Canis lupus familiaris*]	−0.302	0.151
*ADIPOQ*	15825495	3	adiponectin, partial [*Canis lupus familiaris*]	−0.302	0.151
*ADIPOQ*	204638075	3	adiponectin, partial [*Canis lupus*]	−0.302	0.151
*APOA1*	1418241889	5	apolipoprotein A-I [*Canis lupus dingo*]	−0.385	0.151
*APOB*	78499413	5	apolipoprotein B, partial [*Canis mesomelas*]	0.133	0.151
*APOB*	78499415	5	apolipoprotein B, partial [*Canis simensis*]	0.133	0.151
*APOC2*	1418304648	3	apolipoprotein C-II [*Canis lupus dingo*]	−0.395	0.151
*APOC2*	163903	3	apolipoprotein C-II precursor [*Canis lupus familiaris*]	−0.395	0.151
*APOC3*	163905	2	apolipoprotein C-III precursor [*Canis lupus familiaris*]	−0.429	0.151
*APOC3*	924859480	3	apolipoprotein C-III precursor [*Canis lupus familiaris*]	−0.396	0.151
*C3*	1418218430	8	complement C3 [*Canis lupus dingo*]	0.228	0.151
*CFI*	1418243013	10	complement factor I isoform X1 [*Canis lupus dingo*]	−0.093	0.151
*CFI*	1239976293	10	complement factor I isoform X1 [*Canis lupus familiaris*]	−0.093	0.151
*CFI*	1418243015	10	complement factor I isoform X2 [*Canis lupus dingo*]	−0.093	0.151
*CFI*	1239976295	10	complement factor I isoform X2 [*Canis lupus familiaris*]	−0.093	0.151
*CFI*	1418243017	10	complement factor I isoform X3 [*Canis lupus dingo*]	−0.093	0.151
*CFI*	545552242	10	complement factor I isoform X3 [*Canis lupus familiaris*]	−0.093	0.151
*CFI*	1418243019	9	complement factor I isoform X4 [*Canis lupus dingo*]	−0.090	0.151
*CFI*	928179004	9	complement factor I isoform X4 [*Canis lupus familiaris*]	−0.090	0.151
*HPX*	73988725	15	hemopexin [*Canis lupus familiaris*]	0.285	0.151
*ITIH1*	1418216242	21	inter-alpha-trypsin inhibitor heavy chain H1 [*Canis lupus dingo*]	−0.152	0.151
*ITIH1*	73985485	21	inter-alpha-trypsin inhibitor heavy chain H1 isoform X1 [*Canis lupus familiaris*]	−0.152	0.151
*ITIH1*	1239949881	20	inter-alpha-trypsin inhibitor heavy chain H1 isoform X2 [*Canis lupus familiaris*]	−0.155	0.151
*ITIH3*	1418216228	4	inter-alpha-trypsin inhibitor heavy chain H3 isoform X1 [*Canis lupus dingo*]	−0.372	0.151
*ITIH3*	1418216230	4	inter-alpha-trypsin inhibitor heavy chain H3 isoform X2 [*Canis lupus dingo*]	−0.372	0.151
*KRT10*	1418337563	6	keratin, type I cytoskeletal 10 isoform X1 [*Canis lupus dingo*]	−0.308	0.151
*KRT10*	928144438	6	keratin, type I cytoskeletal 10 isoform X1 [*Canis lupus familiaris*]	−0.308	0.151
*KRT10*	1418337565	6	keratin, type I cytoskeletal 10 isoform X2 [*Canis lupus dingo*]	−0.308	0.151
*KRT10*	928144440	6	keratin, type I cytoskeletal 10 isoform X2 [*Canis lupus familiaris*]	−0.308	0.151
*KRT10*	1418337567	6	keratin, type I cytoskeletal 10 isoform X3 [*Canis lupus dingo*]	−0.308	0.151
*KRT10*	928144442	6	keratin, type I cytoskeletal 10 isoform X3 [*Canis lupus familiaris*]	−0.308	0.151
*ITIH3*	928186331	4	LOW QUALITY PROTEIN: inter-alpha-trypsin inhibitor heavy chain H3 [*Canis lupus familiaris*]	−0.372	0.151
*TF*	928167632	3	serotransferrin [*Canis lupus familiaris*]	−0.104	0.151
*LOC112676265*	1418260227	2	uncharacterized protein LOC112676265 [*Canis lupus dingo*]	−0.505	0.151
*HBB*	227343817	14	chain B, crystal structure of dog (*Canis Familiaris*) hemoglobin	1.053	0.190
*HBA*	194368499	2	chain C, hemoglobin subunit alpha	1.059	0.190
*HBD*	103484123	3	globin, partial [*Canis lupus familiaris*]	1.392	0.190
*HBB*	1418222274	13	hemoglobin subunit beta [*Canis lupus dingo*]	1.068	0.190
*HBB*	1418222276	8	hemoglobin subunit beta-like [*Canis lupus dingo*]	1.155	0.190
*MGAM*	1418324508	32	maltase-glucoamylase, intestinal isoform X1 [*Canis lupus dingo*]	0.233	0.190
*MGAM*	1239938912	32	maltase-glucoamylase, intestinal isoform X1 [*Canis lupus familiaris*]	0.233	0.190
*MGAM*	1418324514	32	maltase-glucoamylase, intestinal isoform X2 [*Canis lupus dingo*]	0.233	0.190
*MGAM*	928156540	32	maltase-glucoamylase, intestinal isoform X2 [*Canis lupus familiaris*]	0.233	0.190
*HBA1*	1101972892	9	TPA: globin A1 [*Canis lupus familiaris*]	1.152	0.190
*TFRC*	10946310	8	transferrin receptor [*Canis lupus familiaris*]	−0.296	0.190
*TFRC*	701217752	8	transferrin receptor protein 1 [*Canis lupus*]	−0.296	0.190
*TFRC*	387178037	8	transferrin receptor protein 1 [*Canis mesomelas*]	−0.296	0.190
*MBL2*	1418327084	2	mannose-binding protein C [*Canis lupus dingo*]	−0.640	0.200
*MBL2*	73953733	2	mannose-binding protein C [*Canis lupus familiaris*]	−0.640	0.200
*APOB*	78709133	8	apolipoprotein B, partial [*Canis adustus*]	0.375	0.206
*APOB*	78709135	8	apolipoprotein B, partial [*Canis aureus*]	0.375	0.206
*APOB*	78709137	7	apolipoprotein B, partial [*Canis latrans*]	0.375	0.206
*APOB*	78709143	8	apolipoprotein B, partial [*Canis simensis*]	0.375	0.206
*A1BG*	1418297237	13	alpha-1B-glycoprotein [*Canis lupus dingo*]	−0.165	0.222
*A1BG*	545487024	13	alpha-1B-glycoprotein [*Canis lupus familiaris*]	−0.165	0.222
*APOB*	78709141	6	apolipoprotein B, partial [*Canis mesomelas*]	0.435	0.222
*CP*	1418510222	26	ceruloplasmin isoform X1 [*Canis lupus dingo*]	0.141	0.222
*CP*	1418510226	26	ceruloplasmin isoform X2 [*Canis lupus dingo*]	0.141	0.222
*CP*	1418510228	26	ceruloplasmin isoform X3 [*Canis lupus dingo*]	0.141	0.222
*CP*	1418510230	26	ceruloplasmin isoform X4 [*Canis lupus dingo*]	0.141	0.222
*FN1*	1418343247	57	fibronectin isoform X1 [*Canis lupus dingo*]	−0.124	0.222
*FN1*	928182521	57	fibronectin isoform X1 [*Canis lupus familiaris*]	−0.124	0.222
*FN1*	1418343265	56	fibronectin isoform X10 [*Canis lupus dingo*]	−0.123	0.222
*FN1*	928182523	56	fibronectin isoform X10 [*Canis lupus familiaris*]	−0.123	0.222
*FN1*	1418343267	57	fibronectin isoform X11 [*Canis lupus dingo*]	−0.116	0.222
*FN1*	928182519	57	fibronectin isoform X11 [*Canis lupus familiaris*]	−0.116	0.222
*FN1*	1418343249	57	fibronectin isoform X2 [*Canis lupus dingo*]	−0.124	0.222
*FN1*	1239982239	57	fibronectin isoform X2 [*Canis lupus familiaris*]	−0.124	0.222
*FN1*	1418343251	57	fibronectin isoform X3 [*Canis lupus dingo*]	−0.124	0.222
*FN1*	1239982241	57	fibronectin isoform X3 [*Canis lupus familiaris*]	−0.124	0.222
*FN1*	1418343253	58	fibronectin isoform X4 [*Canis lupus dingo*]	−0.116	0.222
*FN1*	928182507	58	fibronectin isoform X4 [*Canis lupus familiaris*]	−0.116	0.222
*FN1*	1418343255	58	fibronectin isoform X5 [*Canis lupus dingo*]	−0.116	0.222
*FN1*	928182509	58	fibronectin isoform X5 [*Canis lupus familiaris*]	−0.116	0.222
*FN1*	1418343257	56	fibronectin isoform X6 [*Canis lupus dingo*]	−0.123	0.222
*FN1*	1239982243	56	fibronectin isoform X6 [*Canis lupus familiaris*]	−0.123	0.222
*FN1*	1418343259	58	fibronectin isoform X7 [*Canis lupus dingo*]	−0.116	0.222
*FN1*	928182511	58	fibronectin isoform X7 [*Canis lupus familiaris*]	−0.116	0.222
*FN1*	1418343261	54	fibronectin isoform X8 [*Canis lupus dingo*]	−0.108	0.222
*FN1*	1239982245	54	fibronectin isoform X8 [*Canis lupus familiaris*]	−0.108	0.222
*FN1*	1418343263	58	fibronectin isoform X9 [*Canis lupus dingo*]	−0.116	0.222
*FN1*	928182513	58	fibronectin isoform X9 [*Canis lupus familiaris*]	−0.116	0.222
*LOC611458*	1418213976	58	pregnancy zone protein-like isoform X1 [*Canis lupus dingo*]	−0.209	0.222
*LOC611458*	545546412	57	pregnancy zone protein-like isoform X1 [*Canis lupus familiaris*]	−0.204	0.222
*LOC611458*	1418213978	59	pregnancy zone protein-like isoform X2 [*Canis lupus dingo*]	−0.205	0.222
*LOC611458*	545546414	58	pregnancy zone protein-like isoform X2 [*Canis lupus familiaris*]	−0.201	0.222
*LOC611458*	1418213980	51	pregnancy zone protein-like isoform X3 [*Canis lupus dingo*]	−0.177	0.222
*LOC611458*	1239967247	50	pregnancy zone protein-like isoform X3 [*Canis lupus familiaris*]	−0.172	0.222
*CLEC3B*	1418220357	5	tetranectin [*Canis lupus dingo*]	−0.175	0.222
*CLEC3B*	928162811	3	tetranectin [*Canis lupus familiaris*]	−0.197	0.222
*F13B*	545504208	4	coagulation factor XIII B chain [*Canis lupus familiaris*]	−0.209	0.229
*F10*	3328199	4	coagulation factor X, partial [*Canis lupus familiaris*]	0.093	0.257
*F10*	1418340495	4	coagulation factor X-like isoform X1 [*Canis lupus dingo*]	0.093	0.257
*C8G*	1418266608	2	complement component C8 gamma chain isoform X1 [*Canis lupus dingo*]	0.124	0.286
*C8G*	1239920398	2	complement component C8 gamma chain isoform X1 [*Canis lupus familiaris*]	0.124	0.286
*GPX3*	1418327853	7	glutathione peroxidase 3 [*Canis lupus dingo*]	−0.221	0.286
*TFRC*	1418200084	7	transferrin receptor protein 1 [*Canis lupus dingo*]	−0.280	0.286
*ALB*	6687188	4	albumin [*Canis lupus familiaris*]	−0.606	0.310
*SERPINA3*	73964432	12	alpha-1-antichymotrypsin [*Canis lupus familiaris*]	0.098	0.310
*ALB*	1104685307	4	chain A, serum albumin	−0.606	0.310
*C2*	1239928849	7	complement C2 isoform X1 [*Canis lupus familiaris*]	0.054	0.310
*C2*	1418290462	7	complement C2 isoform X2 [*Canis lupus dingo*]	0.054	0.310
*C2*	1239928853	7	complement C2 isoform X3 [*Canis lupus familiaris*]	0.054	0.310
*C7*	1418329743	15	complement component C7 isoform X3 [*Canis lupus dingo*]	0.061	0.310
*GPLD1*	545554529	13	phosphatidylinositol-glycan-specific phospholipase D isoform X1 [*Canis lupus familiaris*]	0.106	0.310
*GPLD1*	1418204633	13	phosphatidylinositol-glycan-specific phospholipase D isoform X2 [*Canis lupus dingo*]	0.106	0.310
*APOD*	928180090	4	apolipoprotein D [*Canis lupus familiaris*]	−0.075	0.341
*CLU*	163954	14	glycoprotein 80 [*Canis lupus familiaris*]	0.102	0.341
*ITIH2*	1418502140	18	inter-alpha-trypsin inhibitor heavy chain H2 [*Canis lupus dingo*]	−0.099	0.389
*ITIH2*	73949158	18	inter-alpha-trypsin inhibitor heavy chain H2 [*Canis lupus familiaris*]	−0.099	0.389
*LBP*	345789637	6	lipopolysaccharide-binding protein [*Canis lupus familiaris*]	0.439	0.400
*LBP*	1418251736	5	LOW QUALITY PROTEIN: lipopolysaccharide-binding protein [*Canis lupus dingo*]	0.498	0.400
*SERPINA3*	1418345970	11	alpha-1-antichymotrypsin [*Canis lupus dingo*]	0.113	0.413
*SAA1*	227017	3	amyloid A protein	−2.012	0.413
*IGJ; JCHAIN*	1239965532	2	immunoglobulin iota chain-like [*Canis lupus familiaris*]	−0.095	0.413
*SAA1*	928166207	4	serum amyloid A protein [*Canis lupus familiaris*]	−1.497	0.413
*SAA1*	1418223200	4	serum amyloid A protein-like [*Canis lupus dingo*]	−1.497	0.413
*SAA1*	57102730	3	serum amyloid A protein-like [*Canis lupus familiaris*]	−2.012	0.413
*SAA1*	55741727	3	serum amyloid A1 precursor [*Canis lupus familiaris*]	−2.012	0.413
*SERPINF2*	1418267349	5	alpha-2-antiplasmin isoform X1 [*Canis lupus dingo*]	0.084	0.421
*SERPINF2*	73967363	5	alpha-2-antiplasmin isoform X1 [*Canis lupus familiaris*]	0.084	0.421
*SERPINF2*	1418267343	5	alpha-2-antiplasmin isoform X2 [*Canis lupus dingo*]	0.084	0.421
*SERPINF2*	545512145	5	alpha-2-antiplasmin isoform X2 [*Canis lupus familiaris*]	0.084	0.421
*APOC1*	1418304654	4	apolipoprotein C-I [*Canis lupus dingo*]	−0.211	0.421
*C4BPB*	1418315459	3	C4b-binding protein beta chain [*Canis lupus dingo*]	−0.082	0.421
*C9*	1418330062	6	complement component C9 [*Canis lupus dingo*]	0.103	0.421
*C9*	545496317	6	complement component C9 [*Canis lupus familiaris*]	0.103	0.421
*FETUB*	74003556	3	fetuin-B [*Canis lupus familiaris*]	0.626	0.421
*HABP2*	1418245880	3	hyaluronan-binding protein 2 [*Canis lupus dingo*]	−0.134	0.421
*HABP2*	545547980	3	hyaluronan-binding protein 2 [*Canis lupus familiaris*]	−0.134	0.421
*ITIH1*	545533419	17	inter-alpha-trypsin inhibitor heavy chain H1 isoform X3 [*Canis lupus familiaris*]	−0.122	0.421
*GPLD1*	928181234	11	phosphatidylinositol-glycan-specific phospholipase D isoform X3 [*Canis lupus familiaris*]	0.129	0.421
*SERPINA5*	1418346186	6	plasma serine protease inhibitor [*Canis lupus dingo*]	0.191	0.421
*SERPINA5*	545508405	6	plasma serine protease inhibitor [*Canis lupus familiaris*]	0.191	0.421
*PLG*	18139619	13	plasminogen, partial [*Canis lupus familiaris*]	−0.099	0.421
*CFP*	74007356	2	properdin [*Canis lupus familiaris*]	0.172	0.421
*F2*	359321961	19	prothrombin [*Canis lupus familiaris*]	0.055	0.421
*APOE*	3915605	11	Apolipoprotein E	0.210	0.421
*SERPING1*	1239944268	7	plasma protease C1 inhibitor [*Canis lupus familiaris*]	−0.098	0.516
*AFM*	1418328671	26	afamin [*Canis lupus dingo*]	−0.067	0.548
*AFM*	73975389	27	afamin [*Canis lupus familiaris*]	−0.068	0.548
*A2M*	1418213974	47	alpha-2-macroglobulin [*Canis lupus dingo*]	−0.034	0.548
*A2M*	345792424	47	alpha-2-macroglobulin [*Canis lupus familiaris*]	−0.034	0.548
*APOB*	78499407	5	apolipoprotein B, partial [*Canis aureus*]	0.033	0.548
*APOB100*	1418212773	74	apolipoprotein B-100 [*Canis lupus dingo*]	0.078	0.548
*APOB100*	545528321	74	apolipoprotein B-100 [*Canis lupus familiaris*]	0.078	0.548
*APOE*	57036446	10	apolipoprotein E [*Canis lupus familiaris*]	0.211	0.548
*F12*	1418325925	3	coagulation factor XII isoform X1 [*Canis lupus dingo*]	−0.053	0.548
*F12*	73953994	3	coagulation factor XII isoform X1 [*Canis lupus familiaris*]	−0.053	0.548
*F12*	1418325927	3	coagulation factor XII isoform X2 [*Canis lupus dingo*]	−0.053	0.548
*F12*	545495643	3	coagulation factor XII isoform X2 [*Canis lupus familiaris*]	−0.053	0.548
*F12*	1418325929	3	coagulation factor XII isoform X3 [*Canis lupus dingo*]	−0.053	0.548
*F12*	1239898360	3	coagulation factor XII isoform X3 [*Canis lupus familiaris*]	−0.053	0.548
*C7*	1418329739	17	complement component C7 isoform X1 [*Canis lupus dingo*]	0.030	0.548
*C7*	73953824	17	complement component C7 isoform X1 [*Canis lupus familiaris*]	0.030	0.548
*C7*	1418329741	16	complement component C7 isoform X2 [*Canis lupus dingo*]	0.071	0.548
*C7*	1239899370	16	complement component C7 isoform X2 [*Canis lupus familiaris*]	0.071	0.548
*FETUB*	1418515534	3	fetuin-B [*Canis lupus dingo*]	0.146	0.548
*HRG*	1418515616	13	histidine-rich glycoprotein isoform X1 [*Canis lupus dingo*]	−0.303	0.548
*HRG*	545553762	12	histidine-rich glycoprotein isoform X1 [*Canis lupus familiaris*]	−0.304	0.548
*IGJ; JCHAIN*	345779666	5	immunoglobulin J chain [*Canis lupus familiaris*]	−0.081	0.548
*IGL-1*	1418263577	3	immunoglobulin lambda-1 light chain-like [*Canis lupus dingo*]	−0.285	0.548
*QSOX1*	928139154	3	LOW QUALITY PROTEIN: sulfhydryl oxidase 1, partial [*Canis lupus familiaris*]	0.061	0.548
*SERPING1*	1418207941	10	plasma protease C1 inhibitor [*Canis lupus dingo*]	−0.102	0.548
*APOA2*	597500968	5	Apolipoprotein A-II;	−0.138	0.548
*QSOX1*	1418315968	3	sulfhydryl oxidase 1 isoform X1 [*Canis lupus dingo*]	0.061	0.548
*QSOX1*	1418315970	3	sulfhydryl oxidase 1 isoform X2 [*Canis lupus dingo*]	0.061	0.548
*QSOX1*	1418315972	3	sulfhydryl oxidase 1 isoform X3 [*Canis lupus dingo*]	0.061	0.548
*VTN*	1418267888	5	vitronectin [*Canis lupus dingo*]	0.022	0.548
*IGH-CH1*	124390007	3	immunoglobulin heavy chain constant region CH1, partial [*Canis lupus familiaris*]	0.187	0.556
*SAA1*	1418223204	7	serum amyloid A protein [*Canis lupus dingo*]	−1.568	0.556
*SAA1*	545536980	7	serum amyloid A protein isoform X1 [*Canis lupus familiaris*]	−1.568	0.556
*SAA1*	164060	7	serum amyloid A protein, partial [*Canis lupus familiaris*]	−1.568	0.556
*SAA1*	164064	7	serum amyloid A protein, partial [*Canis lupus familiaris*]	−1.568	0.556
*SAA1*	164066	7	serum amyloid A protein, partial [*Canis lupus familiaris*]	−1.568	0.556
*SAA1*	164068	7	serum amyloid A protein, partial [*Canis lupus familiaris*]	−1.568	0.556
*SAA1*	1418223194	7	serum amyloid A protein-like [*Canis lupus dingo*]	−1.568	0.556
*SAA1*	545535766	7	serum amyloid A1 isoform X1 [*Canis lupus familiaris*]	−1.568	0.556
*KRT1*	1418221463	6	keratin, type II cytoskeletal 1 [*Canis lupus dingo*]	−0.250	0.629
*KRT1*	75062693	6	Keratin, type II cytoskeletal 1;	−0.250	0.629
*PEPD*	1418305616	2	xaa-Pro dipeptidase isoform X1 [*Canis lupus dingo*]	−0.179	0.629
*PEPD*	73948526	2	xaa-Pro dipeptidase isoform X1 [*Canis lupus familiaris*]	−0.179	0.629
*PEPD*	545489037	2	xaa-Pro dipeptidase isoform X2 [*Canis lupus familiaris*]	−0.179	0.629
*APOB*	62870505	2	apolipoprotein B, partial [*Canis lupus*]	0.277	0.667
*APOB*	256016957	2	apolipoprotein B, partial [*Canis lupus*]	0.277	0.667
*APOB*	269307499	2	apolipoprotein B, partial [*Canis lupus*]	0.277	0.667
*APOB100*	925718015	2	apolipoprotein B-100, partial [Canis adustus]	0.277	0.667
*APOB100*	925718021	2	apolipoprotein B-100, partial [*Canis aureus*]	0.277	0.667
*APOB100*	925718027	2	apolipoprotein B-100, partial [*Canis aureus*]	0.277	0.667
*APOB100*	925718029	2	apolipoprotein B-100, partial [*Canis aureus*]	0.277	0.667
*APOB100*	925718033	2	apolipoprotein B-100, partial [*Canis latrans*]	0.277	0.667
*APOB100*	925718059	2	apolipoprotein B-100, partial [*Canis latrans*]	0.277	0.667
*APOB100*	925718053	2	apolipoprotein B-100, partial [*Canis lupus*]	0.277	0.667
*APOB100*	925718037	2	apolipoprotein B-100, partial [*Canis simensis*]	0.277	0.667
*IGHE*	1096665	2	Ig:SUBUNIT = epsilon	−1.005	0.667
*THBS1*	1418259722	4	thrombospondin-1 [*Canis lupus dingo*]	−0.341	0.667
*THBS1*	345794639	4	thrombospondin-1 [*Canis lupus familiaris*]	−0.341	0.667
*LRG1*	1418218016	7	leucine-rich alpha-2-glycoprotein [*Canis lupus dingo*]	0.005	0.683
*SERPINF2*	1418267345	4	alpha-2-antiplasmin isoform X3 [*Canis lupus dingo*]	0.046	0.690
*SERPINF2*	1239920122	4	alpha-2-antiplasmin isoform X3 [*Canis lupus familiaris*]	0.046	0.690
*SERPINF2*	1418267347	4	alpha-2-antiplasmin isoform X4 [*Canis lupus dingo*]	0.046	0.690
*SERPINF2*	1239920124	4	alpha-2-antiplasmin isoform X4 [*Canis lupus familiaris*]	0.046	0.690
*C4BPA*	1418315462	24	C4b-binding protein alpha chain [*Canis lupus dingo*]	0.079	0.690
*CPN1*	1418245069	3	carboxypeptidase N catalytic chain isoform X2 [*Canis lupus dingo*]	0.222	0.690
*SERPINA6*	545508387	3	corticosteroid-binding globulin [*Canis lupus familiaris*]	0.062	0.690
*SERPINA6*	1418346025	3	corticosteroid-binding globulin-like [*Canis lupus dingo*]	0.062	0.690
*IGH*	1340236572	2	immunoglobulin heavy chain variable region, partial [*Canis lupus familiaris*]	−0.176	0.690
*IGJ; JCHAIN*	19715661	2	immunoglobulin J chain, partial [*Canis lupus familiaris*]	−0.130	0.690
*PLG*	558695388	33	plasminogen precursor [*Canis lupus familiaris*]	−0.015	0.690
*ACT*	1418333042	2	actin, clone 302-like [*Canis lupus dingo*]	−0.005	0.700
*ACTB*	1222669	2	beta-actin, partial [*Canis lupus familiaris*]	−0.005	0.700
*N/A*	1418204343	2	C-C motif chemokine 14-like [*Canis lupus dingo*]	−0.109	0.700
*N/A*	1535087818	3	unnamed protein product [*Toxocara canis*]	−0.005	0.700
*AZGP1*	70909945	5	zinc alpha-2-glycoprotein 1, partial [*Canis lupus familiaris*]	−0.016	0.700
*IGH-CH2*	124390009	3	immunoglobulin heavy chain constant region CH2, partial [*Canis lupus familiaris*]	−0.019	0.722
*ORM1*	1418322788	8	alpha-1-acid glycoprotein 1-like [*Canis lupus dingo*]	0.258	0.730
*C3*	1418218853	5	complement C3-like [*Canis lupus dingo*]	−0.274	0.730
*IGL-1*	545544683	3	immunoglobulin lambda-1 light chain isoform X34 [*Canis lupus familiaris*]	−0.156	0.730
*TF*	1418510423	2	serotransferrin-like [*Canis lupus dingo*]	−0.219	0.730
*C1r*	1418256182	10	complement C1r subcomponent [*Canis lupus dingo*]	0.000	0.786
*AHSG*	1418515495	10	alpha-2-HS-glycoprotein [*Canis lupus dingo*]	−0.332	0.841
*APOE4*	283972739	5	apolipoprotein E4, partial [*Canis lupus familiaris*]	0.158	0.841
*APOE4*	283972741	5	apolipoprotein E4, partial [*Canis lupus familiaris*]	0.158	0.841
*APOE4*	283972753	5	apolipoprotein E4, partial [*Canis lupus familiaris*]	0.158	0.841
*APOM*	57094349	3	apolipoprotein M isoform X1 [*Canis lupus familiaris*]	−0.021	0.841
*CD5L*	590121823	15	apoptosis inhibitor of macrophage [*Canis lupus familiaris*]	−0.093	0.841
*CPN2*	545553264	3	carboxypeptidase N subunit 2 [*Canis lupus familiaris*]	0.083	0.841
*CD5L*	1418317684	15	CD5 antigen-like [*Canis lupus dingo*]	−0.093	0.841
*C1s*	73997275	6	complement C1s subcomponent [*Canis lupus familiaris*]	−0.048	0.841
*CFB*	1418290466	27	complement factor B [*Canis lupus dingo*]	−0.039	0.841
*CFB*	345778397	27	complement factor B [*Canis lupus familiaris*]	−0.039	0.841
*HRG*	1418515618	9	histidine-rich glycoprotein isoform X2 [*Canis lupus dingo*]	−0.164	0.841
*HRG*	545553764	9	histidine-rich glycoprotein isoform X2 [*Canis lupus familiaris*]	−0.164	0.841
*IGH-CH4*	124390013	4	immunoglobulin heavy chain constant region CH4, partial [*Canis lupus familiaris*]	−0.280	0.841
*IGH*	208342048	3	immunoglobulin heavy chain variable region, partial [*Canis lupus familiaris*]	0.190	0.841
*ITIH4*	928186325	30	inter-alpha-trypsin inhibitor heavy chain H4 isoform X1 [*Canis lupus familiaris*]	0.002	0.841
*ITIH4*	1239883263	29	inter-alpha-trypsin inhibitor heavy chain H4 isoform X2 [*Canis lupus familiaris*]	0.002	0.841
*ITIH4*	928186327	30	inter-alpha-trypsin inhibitor heavy chain H4 isoform X3 [*Canis lupus familiaris*]	0.002	0.841
*CPN2*	1418198271	3	LOW QUALITY PROTEIN: carboxypeptidase N subunit 2-like [*Canis lupus dingo*]	0.083	0.841
*MGAM2*	1418324502	3	putative maltase-glucoamylase-like protein FLJ16351 [*Canis lupus dingo*]	−0.008	0.841
*MGAM2*	1239938902	3	putative maltase-glucoamylase-like protein FLJ16351 isoform X1 [*Canis lupus familiaris*]	−0.008	0.841
*MGAM2*	1239938904	3	putative maltase-glucoamylase-like protein FLJ16351 isoform X2 [*Canis lupus familiaris*]	−0.008	0.841
*IGH*	208342196	3	immunoglobulin heavy chain variable region, partial [*Canis lupus familiaris*]	0.122	0.857
*SAA1*	545536976	6	serum amyloid A protein-like [*Canis lupus familiaris*]	−1.160	0.886
*CPN1*	928175986	4	carboxypeptidase N catalytic chain isoform X1 [*Canis lupus familiaris*]	−0.001	0.905
*CP*	928167527	25	ceruloplasmin isoform X1 [*Canis lupus familiaris*]	0.060	0.905
*CP*	1239957836	25	ceruloplasmin isoform X2 [*Canis lupus familiaris*]	0.060	0.905
*CP*	73990367	25	ceruloplasmin isoform X3 [*Canis lupus familiaris*]	0.060	0.905
*CP*	1239957839	25	ceruloplasmin isoform X4 [*Canis lupus familiaris*]	0.060	0.905
*C1qA*	1418506879	2	complement C1q subcomponent subunit A [*Canis lupus dingo*]	0.063	0.905
*IGL*	164430486	2	immunoglobulin lambda light chain variable region, partial [*Canis lupus familiaris*]	0.079	0.905
*IGL*	164430516	2	immunoglobulin lambda light chain variable region, partial [*Canis lupus familiaris*]	0.079	0.905
*ADIPOQ*	1483452888	2	TPA: adiponectin C [*Canis lupus familiaris*]	0.063	0.905
*VDB*	1418328657	26	vitamin D-binding protein [*Canis lupus dingo*]	0.012	0.952
*SERPINC1*	359320010	23	antithrombin-III [*Canis lupus familiaris*]	0.044	1.000
*APOA4*	1418241891	25	apolipoprotein A-IV [*Canis lupus dingo*]	−0.057	1.000
*APOA4*	345799905	25	apolipoprotein A-IV [*Canis lupus familiaris*]	−0.057	1.000
*CP*	45826457	5	ceruloplasmin, partial [*Canis lupus familiaris*]	−0.042	1.000
*F13A*	1418205161	2	coagulation factor XIII A chain [*Canis lupus dingo*]	0.063	1.000
*C4A*	1239928570	60	complement C4-A [*Canis lupus familiaris*]	0.053	1.000
*C4A*	1418290456	62	complement C4-A-like [*Canis lupus dingo*]	0.036	1.000
*C8B*	73956394	3	complement component C8 beta chain [*Canis lupus familiaris*]	0.003	1.000
*HP*	258498	4	haptoglobin light chain, HpL chain [dogs, Peptide, 83 aa]	0.127	1.000
*HGFAC*	1418333238	2	hepatocyte growth factor activator [*Canis lupus dingo*]	0.026	1.000
*HGFAC*	1239893961	2	hepatocyte growth factor activator isoform X1 [*Canis lupus familiaris*]	0.026	1.000
*HGFAC*	73532760	2	hepatocyte growth factor activator precursor [*Canis lupus familiaris*]	0.026	1.000
*IGH-CH3*	124390011	2	immunoglobulin heavy chain constant region CH3, partial [*Canis lupus familiaris*]	−0.077	1.000
*IGL*	164430480	2	immunoglobulin lambda light chain variable region, partial [*Canis lupus familiaris*]	0.019	1.000
*IGM*	146743249	3	immunoglobulin mu heavy chain variable region, partial [*Canis lupus familiaris*]	−0.219	1.000
*LUM*	1418336203	2	lumican [*Canis lupus dingo*]	−0.144	1.000
*MFAP4*	1418312041	2	microfibril-associated glycoprotein 4 [*Canis lupus dingo*]	−0.057	1.000
*APOA4*	704000372	21	Apolipoprotein A-IV	−0.054	1.000
*PLG*	130314	16	Plasminogen	0.013	1.000
*PON1*	73975797	13	serum paraoxonase/arylesterase 1 [*Canis lupus familiaris*]	−0.073	1.000
*SHBG*	1418312750	5	sex hormone-binding globulin isoform X1 [*Canis lupus dingo*]	−0.105	1.000
*SHBG*	1239901836	5	sex hormone-binding globulin isoform X2 [*Canis lupus familiaris*]	−0.105	1.000
*TTR*	1418314244	5	transthyretin [*Canis lupus dingo*]	−0.062	1.000
*PROC*	1418199974	16	vitamin K-dependent protein S [*Canis lupus dingo*]	0.024	1.000
*PROC*	1239976411	16	vitamin K-dependent protein S [*Canis lupus familiaris*]	0.024	1.000
*VTN*	62421376	2	vitronectin, partial [*Canis mesomelas*]	−0.013	1.000
*AZGP1*	560879429	8	zinc-alpha-2-glycoprotein precursor [*Canis lupus familiaris*]	0.158	1.000

**Table A2:** Salivary proteins identified in dogs with canine mammary tumors (CMT) and healthy controls (HC).

Gene Name	Accession Number	No. Unique Peptides	Protein Description	Fold Change (log2)	*p*-Value
*LYPD3*	1418304786	4	ly6/PLAUR domain-containing protein 3 [*Canis lupus dingo*]	0.594	0.008
*LYPD3*	73948247	4	ly6/PLAUR domain-containing protein 3 [*Canis lupus familiaris*]	0.594	0.008
*MDH2*	1418307145	4	malate dehydrogenase, mitochondrial [*Canis lupus dingo*]	0.981	0.008
*MDH2*	1239955263	3	malate dehydrogenase, mitochondrial-like [*Canis lupus familiaris*]	1.037	0.008
*MDH2*	89574135	3	mitochondrial malate dehydrogenase 2, NAD, partial [*Canis lupus familiaris*]	0.831	0.008
*NME2*	545510196	3	nucleoside diphosphate kinase B isoform X1 [*Canis lupus familiaris*]	0.722	0.008
*PRDX1*	1418294943	4	peroxiredoxin-1 [*Canis lupus dingo*]	0.834	0.008
*S100A6*	1239912330	3	protein S100-A6 [*Canis lupus familiaris*]	1.236	0.008
*TPI1*	76363530	12	Triosephosphate isomerase	0.597	0.008
*CAP1*	1418293878	8	adenylyl cyclase-associated protein 1 isoform X1 [*Canis lupus dingo*]	0.795	0.016
*CAP1*	1418293884	8	adenylyl cyclase-associated protein 1 isoform X2 [*Canis lupus dingo*]	0.795	0.016
*ANXA1*	558695394	9	annexin A1 [*Canis lupus familiaris*]	0.754	0.016
*CALM3*	1418304395	2	calmodulin-3 isoform X2 [*Canis lupus dingo*]	0.531	0.016
*CORO1A*	1418213540	2	cornifin-A [*Canis lupus dingo*]	0.890	0.016
*EZR*	1418300273	7	ezrin isoform X2 [*Canis lupus dingo*]	0.437	0.016
*KLK1*	1418303522	3	kallikrein-1-like isoform X1 [*Canis lupus dingo*]	−0.846	0.016
*KLK1*	1418303524	3	kallikrein-1-like isoform X2 [*Canis lupus dingo*]	−0.846	0.016
*CANF1*	1418264299	13	major allergen Can f 1 [*Canis lupus dingo*]	1.211	0.016
*MDH1*	57092971	3	malate dehydrogenase, cytoplasmic [*Canis lupus familiaris*]	0.655	0.016
*MDH2*	1418335689	2	malate dehydrogenase, mitochondrial-like [*Canis lupus dingo*]	0.943	0.016
*CANF1*	3121745	13	Major allergen Can f 1	1.211	0.016
*XDH*	1418212080	7	xanthine dehydrogenase/oxidase isoform X1 [*Canis lupus dingo*]	0.610	0.016
*XDH*	73980076	7	xanthine dehydrogenase/oxidase isoform X1 [*Canis lupus familiaris*]	0.610	0.016
*XDH*	1418212082	7	xanthine dehydrogenase/oxidase isoform X2 [*Canis lupus dingo*]	0.610	0.016
*XDH*	545527502	7	xanthine dehydrogenase/oxidase isoform X2 [*Canis lupus familiaris*]	0.610	0.016
*ACTN4*	1418305407	13	alpha-actinin-4 isoform X1 [*Canis lupus dingo*]	0.619	0.032
*ACTN4*	1418305409	12	alpha-actinin-4 isoform X2 [*Canis lupus dingo*]	0.619	0.032
*ACTN4*	1418305411	13	alpha-actinin-4 isoform X3 [*Canis lupus dingo*]	0.619	0.032
*ACTN4*	73947736	12	alpha-actinin-4 isoform X4 [*Canis lupus familiaris*]	0.605	0.032
*ACTN4*	1418305415	11	alpha-actinin-4 isoform X5 [*Canis lupus dingo*]	0.603	0.032
*CALM2*	1418197013	2	calmodulin-2 [*Canis lupus dingo*]	0.566	0.032
*NPC2*	945179	3	CE1 [*Canis lupus familiaris*]	0.358	0.032
*EZR*	558757359	9	ezrin [*Canis lupus familiaris*]	0.413	0.032
*NPC2*	356582247	3	NPC intracellular cholesterol transporter 2 precursor [*Canis lupus familiaris*]	0.358	0.032
*PPIA*	1418202786	2	peptidyl-prolyl cis-trans isomerase A isoform X2 [*Canis lupus dingo*]	0.608	0.032
*UBB*	1418312167	3	polyubiquitin-B [*Canis lupus dingo*]	0.735	0.032
*UBB*	1239902229	3	polyubiquitin-B [*Canis lupus familiaris*]	0.735	0.032
*UBC*	1418260846	3	polyubiquitin-C [*Canis lupus dingo*]	0.735	0.032
*UBC*	73995130	3	polyubiquitin-C [*Canis lupus familiaris*]	0.735	0.032
*PDIA3*	1418259105	4	protein disulfide-isomerase A3 [*Canis lupus dingo*]	0.405	0.032
*S100A2*	1418313584	5	protein S100-A2 [*Canis lupus dingo*]	1.006	0.032
*S100A4*	1391723726	3	protein S100-A4 isoform 1 [*Canis lupus familiaris*]	1.123	0.032
*S100A4*	1473222592	3	protein S100-A4 isoform 2 [*Canis lupus familiaris*]	1.123	0.032
*UBA52*	1418311328	3	ubiquitin [*Canis lupus dingo*]	0.735	0.032
*UBA52*	5822852	3	ubiquitin, partial [*Canis lupus familiaris*]	0.735	0.032
*UBA52*	356582340	3	ubiquitin-40S ribosomal protein S27a [*Canis lupus familiaris*]	0.735	0.032
*UBB*	1239889921	2	ubiquitin-like [*Canis lupus familiaris*]	0.769	0.032
*UBA52*	5441519	3	ubiquitin-ribosomal protein L40 fusion protein [*Canis lupus familiaris*]	0.735	0.032
*MUC5B*	1418206782	2	LOW QUALITY PROTEIN: mucin-5B [*Canis lupus dingo*]	0.583	0.040
*YWHAE*	73967156	5	14-3-3 protein epsilon isoform X2 [*Canis lupus familiaris*]	0.684	0.056
*ARPC2*	1418341783	5	actin-related protein 2/3 complex subunit 2 [*Canis lupus dingo*]	0.763	0.056
*ARPC4*	928162268	4	actin-related protein 2/3 complex subunit 4 [*Canis lupus familiaris*]	1.028	0.056
*ENO1*	1418310054	15	alpha-enolase isoform X2 [*Canis lupus dingo*]	0.711	0.056
*GLUL*	158430851	2	chain A, glutamine synthetase	0.839	0.056
	1418503128	2	elongation factor 1-gamma [*Canis lupus dingo*]	0.399	0.056
	1418314796	2	elongation factor 1-gamma-like [*Canis lupus dingo*]	0.399	0.056
*FLNA*	74008809	6	filamin-A [*Canis lupus familiaris*]	0.850	0.056
*FLNA*	1418225135	6	filamin-A isoform X1 [*Canis lupus dingo*]	0.850	0.056
*FLNA*	1418225137	6	filamin-A isoform X2 [*Canis lupus dingo*]	0.850	0.056
*ALDOA*	1418280689	8	fructose-bisphosphate aldolase A [*Canis lupus dingo*]	0.672	0.056
*GLUL*	648216199	2	glutamine synthetase isoform 1 [*Canis lupus familiaris*]	0.839	0.056
*GLUL*	648216006	2	glutamine synthetase isoform 2 [*Canis lupus familiaris*]	0.839	0.056
*HSP90AA1*	1418344784	10	heat shock protein HSP 90-alpha [*Canis lupus dingo*]	0.625	0.056
*HSP90AA1*	928142969	8	heat shock protein HSP 90-alpha [*Canis lupus familiaris*]	0.625	0.056
*PPIA*	1239883019	3	peptidyl-prolyl cis-trans isomerase A [*Canis lupus familiaris*]	0.447	0.056
*S100A11*	928158750	2	protein S100-A11 [*Canis lupus familiaris*]	0.994	0.056
*S100A11*	1418213478	2	protein S100-A11, partial [*Canis lupus dingo*]	0.994	0.056
*S100A12*	1418313614	8	protein S100-A12-like [*Canis lupus dingo*]	1.044	0.056
*S100A9*	928140605	9	protein S100-A9 [*Canis lupus familiaris*]	1.322	0.056
*S100A8*	224969390	7	S100 calcium-binding protein A8 [*Canis lupus familiaris*]	1.215	0.056
*SPINK5*	1418507526	4	serine protease inhibitor Kazal-type 5 [*Canis lupus dingo*]	0.439	0.056
*SPINK5*	70794744	4	serine protease inhibitor Kazal-type 5 precursor [*Canis lupus familiaris*]	0.439	0.056
*TKT*	545533393	25	transketolase [*Canis lupus familiaris*]	0.608	0.056
*TKT*	1418216286	25	transketolase isoform X1 [*Canis lupus dingo*]	0.608	0.056
*TKT*	1418216288	25	transketolase isoform X2 [*Canis lupus dingo*]	0.608	0.056
*CDH1*	1418308718	3	cadherin-1 [*Canis lupus dingo*]	0.767	0.057
*DPYD*	1418288800	2	dihydropyrimidine dehydrogenase [NADP(+)] isoform X1 [*Canis lupus dingo*]	0.807	0.057
*DPYD*	1418288802	2	dihydropyrimidine dehydrogenase [NADP(+)] isoform X2 [*Canis lupus dingo*]	0.807	0.057
*DPYD*	1239908722	2	dihydropyrimidine dehydrogenase [NADP(+)] isoform X2 [*Canis lupus familiaris*]	0.807	0.057
*N/A*	1239891563	4	double-headed protease inhibitor, submandibular gland [*Canis lupus familiaris*]	0.236	0.057
*VCL*	305657831	3	metavinculin variant, partial [*Canis lupus familiaris*]	1.340	0.057
*PNP*	1418335936	3	purine nucleoside phosphorylase [*Canis lupus dingo*]	0.488	0.057
*NPEPPS*	1418337649	3	puromycin-sensitive aminopeptidase isoform X1 [*Canis lupus dingo*]	0.827	0.057
*NPEPPS*	545511022	3	puromycin-sensitive aminopeptidase isoform X1 [*Canis lupus familiaris*]	0.827	0.057
*NPEPPS*	1418337651	3	puromycin-sensitive aminopeptidase isoform X2 [*Canis lupus dingo*]	0.827	0.057
*NPEPPS*	1418337655	3	puromycin-sensitive aminopeptidase isoform X4 [*Canis lupus dingo*]	0.827	0.057
*CDH1*	550600200	3	Cadherin-1E-Cad/CTF1;	0.767	0.057
*VCL*	345798988	5	vinculin [*Canis lupus familiaris*]	1.280	0.057
*VCL*	1418326635	5	vinculin isoform X1 [*Canis lupus dingo*]	1.280	0.057
*VCL*	1418326637	5	vinculin isoform X2 [*Canis lupus dingo*]	1.280	0.057
*CORO1A*	345801939	5	coronin-1A [*Canis lupus familiaris*]	0.693	0.063
*ARHGDIB*	57106959	7	rho GDP-dissociation inhibitor 2 [*Canis lupus familiaris*]	0.511	0.063
*VCP*	1418320363	2	transitional endoplasmic reticulum ATPase [*Canis lupus dingo*]	0.450	0.063
*VCP*	1239927670	2	transitional endoplasmic reticulum ATPase isoform X2 [*Canis lupus familiaris*]	0.450	0.063
*ACSS1*	1418227068	2	acetyl-coenzyme A synthetase 2-like, mitochondrial [*Canis lupus dingo*]	0.774	0.086
*ACSS1*	359322579	2	acetyl-coenzyme A synthetase 2-like, mitochondrial [*Canis lupus familiaris*]	0.774	0.086
*ACTR3*	1418202110	4	actin-related protein 3 [*Canis lupus dingo*]	0.816	0.095
*ACTR3*	345784150	4	actin-related protein 3 [*Canis lupus familiaris*]	0.816	0.095
*ENO1*	1418310052	17	alpha-enolase isoform X1 [*Canis lupus dingo*]	0.626	0.095
*ENO3*	928134045	4	beta-enolase isoform X1 [*Canis lupus familiaris*]	0.845	0.095
*ENO3*	1418313159	4	beta-enolase isoform X2 [*Canis lupus dingo*]	0.845	0.095
*CD177*	1418304784	3	CD177 antigen [*Canis lupus dingo*]	0.732	0.095
*GSTA4*	1418292151	3	glutathione S-transferase A4-like [*Canis lupus dingo*]	0.750	0.095
*GSTA4*	1239929974	3	glutathione S-transferase A4-like [*Canis lupus familiaris*]	0.750	0.095
*GSTM1*	1418288349	11	glutathione S-transferase Mu 1 isoform X1 [*Canis lupus dingo*]	0.321	0.095
*HSPA4*	1239925350	5	heat shock 70 kDa protein 4 isoform X1 [*Canis lupus familiaris*]	0.538	0.095
*HSPA4*	545516431	5	heat shock 70 kDa protein 4 isoform X2 [*Canis lupus familiaris*]	0.538	0.095
*HSPA4*	1418322757	5	heat shock 70 kDa protein 4 isoform X3 [*Canis lupus dingo*]	0.538	0.095
*HSPA8*	545497049	8	heat shock cognate 71 kDa protein [*Canis lupus familiaris*]	0.629	0.095
*HSPA4*	62631867	5	heat shock protein Apg-2 [*Canis lupus familiaris*]	0.538	0.095
*HISTH4*	1418253529	3	histone H4-like [*Canis lupus dingo*]	0.857	0.095
*LDHA*	1418223224	10	L-lactate dehydrogenase A chain isoform X1 [*Canis lupus dingo*]	0.601	0.095
*LDHA*	545536994	10	L-lactate dehydrogenase A chain isoform X2 [*Canis lupus familiaris*]	0.601	0.095
*ENO1*	345800677	14	LOW QUALITY PROTEIN: alpha-enolase [*Canis lupus familiaris*]	0.676	0.095
*ENO1*	928125111	13	LOW QUALITY PROTEIN: alpha-enolase-like [*Canis lupus familiaris*]	0.627	0.095
*CD177*	1239888116	3	LOW QUALITY PROTEIN: CD177 antigen [*Canis lupus familiaris*]	0.732	0.095
*LOC106558262*	1239979503	3	LOW QUALITY PROTEIN: uncharacterized protein LOC106558262 [*Canis lupus familiaris*]	0.857	0.095
*LOC112674420*	1418253519	3	LOW QUALITY PROTEIN: uncharacterized protein LOC112674420 [*Canis lupus dingo*]	0.857	0.095
*LOC488306*	928180958	3	LOW QUALITY PROTEIN: uncharacterized protein LOC488306 [*Canis lupus familiaris*]	0.857	0.095
*LCP1*	1239955970	17	plastin-2 isoform X1 [*Canis lupus familiaris*]	0.573	0.095
*LCP1*	545537421	17	plastin-2 isoform X2 [*Canis lupus familiaris*]	0.573	0.095
*PLS3*	1239983604	12	plastin-3 [*Canis lupus familiaris*]	0.390	0.095
*PLS3*	1418318928	12	plastin-3 isoform X1 [*Canis lupus dingo*]	0.390	0.095
*TF*	928167632	2	serotransferrin [*Canis lupus familiaris*]	0.480	0.095
*KLK1*	264597	2	tissue kallikrein A beta-chain, CPK-A beta-chain {N-terminal} {EC 3.4.21.35} [dogs, pancreas, peptide partial, 38 aa]	−0.830	0.095
*KLK1*	264599	2	tissue kallikrein B beta-chain, CPK-B beta-chain {N-terminal} {EC 3.4.21.35} [dogs, pancreas, peptide partial, 39 aa]	−0.830	0.095
*LOC100856160*	928181068	3	uncharacterized protein LOC100856160 [*Canis lupus familiaris*]	0.857	0.095
*LOC112674397*	1418253487	3	uncharacterized protein LOC112674397 isoform X2 [*Canis lupus dingo*]	0.857	0.095
*LOC112674463*	1418253569	3	uncharacterized protein LOC112674463 [*Canis lupus dingo*]	0.857	0.095
*AHCY*	73991635	3	adenosylhomocysteinase [*Canis lupus familiaris*]	0.653	0.100
*CALM1*	1239893625	2	calmodulin, partial [*Canis lupus familiaris*]	0.718	0.100
*APOA1*	1418241889	15	apolipoprotein A-I [*Canis lupus dingo*]	0.813	0.111
*PTMA*	1418208278	2	prothymosin alpha [*Canis lupus dingo*]	1.138	0.111
*PTMA*	1418231166	2	prothymosin alpha [*Canis lupus dingo*]	1.138	0.111
*PTMA*	356582259	2	prothymosin alpha [*Canis lupus familiaris*]	1.138	0.111
*ARPC2*	1239981819	4	actin-related protein 2/3 complex subunit 2 [*Canis lupus familiaris*]	1.147	0.114
*EEF2*	1418240337	6	elongation factor 2 [*Canis lupus dingo*]	0.757	0.114
*PSAP*	545495176	8	prosaposin isoform X1 [*Canis lupus familiaris*]	0.188	0.143
*PSAP*	545495178	8	prosaposin isoform X2 [*Canis lupus familiaris*]	0.188	0.143
*PSAP*	73952852	8	prosaposin isoform X3 [*Canis lupus familiaris*]	0.188	0.143
*PSAP*	545495181	8	prosaposin isoform X4 [*Canis lupus familiaris*]	0.188	0.143
*PSAP*	545495183	8	prosaposin isoform X5 [*Canis lupus familiaris*]	0.188	0.143
*PSAP*	1418325065	8	prosaposin isoform X6 [*Canis lupus dingo*]	0.188	0.143
*YWHAE*	1418267412	4	14-3-3 protein epsilon isoform X1 [*Canis lupus dingo*]	0.741	0.151
*YWHAE*	1418267416	3	14-3-3 protein epsilon isoform X3 [*Canis lupus dingo*]	0.736	0.151
*YWHAE*	1418267418	3	14-3-3 protein epsilon isoform X4 [*Canis lupus dingo*]	0.736	0.151
*YWHAQ*	1418213116	3	14-3-3 protein theta [*Canis lupus dingo*]	0.531	0.151
*ARPC3*	1239964305	2	actin-related protein 2/3 complex subunit 3 isoform X1 [*Canis lupus familiaris*]	0.811	0.151
*ARPC3*	1418261548	2	actin-related protein 2/3 complex subunit 3 isoform X2 [*Canis lupus dingo*]	0.811	0.151
*ARPC3*	1418261550	2	actin-related protein 2/3 complex subunit 3 isoform X3 [*Canis lupus dingo*]	0.811	0.151
*ANGPTL5*	545514351	5	angiopoietin-related protein 5-like [*Canis lupus familiaris*]	−0.744	0.151
*CSTM*	1418503443	3	cystatin-M [*Canis lupus dingo*]	−0.757	0.151
*DMBT1*	928186547	3	deleted in malignant brain tumors 1 protein-like, partial [*Canis lupus familiaris*]	−0.479	0.151
*FGG*	545524897	6	fibrinogen gamma chain isoform X1 [*Canis lupus familiaris*]	0.613	0.151
*FGG*	73977992	6	fibrinogen gamma chain isoform X2 [*Canis lupus familiaris*]	0.613	0.151
*HPX*	73988725	7	hemopexin [*Canis lupus familiaris*]	0.612	0.151
*HISTH4*	50542205	2	histone H4, partial [*Canis lupus familiaris*]	0.794	0.151
*CAPG*	1418211040	6	macrophage-capping protein isoform X1 [*Canis lupus dingo*]	0.502	0.151
*CAPG*	545528000	6	macrophage-capping protein isoform X1 [*Canis lupus familiaris*]	0.502	0.151
*MYH9*	1418195836	21	myosin-9 [*Canis lupus dingo*]	0.566	0.151
*MMP8*	345799783	4	neutrophil collagenase [*Canis lupus familiaris*]	0.349	0.151
*ELANE*	1418220639	4	neutrophil elastase [*Canis lupus dingo*]	0.652	0.151
*ELANE*	50979246	4	neutrophil elastase precursor [*Canis lupus familiaris*]	0.652	0.151
*PTGR1*	1418321329	6	prostaglandin reductase 1 [*Canis lupus dingo*]	0.644	0.151
*PTGR1*	545517912	6	prostaglandin reductase 1 [*Canis lupus familiaris*]	0.644	0.151
*MYH9*	122135145	21	Myosin-9	0.566	0.151
*ARHGDIA*	73964747	3	rho GDP-dissociation inhibitor 1 [*Canis lupus familiaris*]	0.486	0.151
*SERPINC1*	359320010	2	antithrombin-III [*Canis lupus familiaris*]	0.800	0.190
*GSTM1*	1239908416	10	glutathione S-transferase Mu 1 isoform X2 [*Canis lupus familiaris*]	0.244	0.190
*HP*	258499	14	haptoglobin heavy chain, HpH chain [dogs, Peptide, 245 aa]	0.445	0.190
*HP*	73957095	16	haptoglobin-like [*Canis lupus familiaris*]	0.479	0.190
*LEG1*	1418301038	7	protein LEG1 homolog [*Canis lupus dingo*]	0.414	0.190
*LEG1*	1239885884	7	protein LEG1 homolog [*Canis lupus familiaris*]	0.414	0.190
*APOA1*	3915607	13	Apolipoprotein A-I	0.808	0.190
*HP*	123511	16	Haptoglobin	0.479	0.190
*S100P*	345798353	2	protein S100-P [*Canis lupus familiaris*]	0.598	0.198
*KRT15*	1418338550	4	keratin, type I cytoskeletal 15 [*Canis lupus dingo*]	0.430	0.200
*SERPINB10*	1418298925	2	serpin B10 [*Canis lupus dingo*]	0.035	0.200
*SERPINB10*	73945839	2	serpin B10 [*Canis lupus familiaris*]	0.035	0.200
*YWHAZ*	928151832	8	14-3-3 protein zeta/delta [*Canis lupus familiaris*]	0.445	0.222
*YWHAZ*	1418511015	7	14-3-3 protein zeta/delta-like [*Canis lupus dingo*]	0.494	0.222
*ACTB*	924183513	4	actin, cytoplasmic 1 [*Canis lupus familiaris*]	−0.283	0.222
*ACTB*	545500782	4	actin, cytoplasmic 1 isoform X1 [*Canis lupus familiaris*]	−0.283	0.222
*ACTB*	1418248118	4	actin, cytoplasmic 2 [*Canis lupus dingo*]	−0.283	0.222
*ACTG1*	924442847	4	actin, cytoplasmic 2 [*Canis lupus familiaris*]	−0.283	0.222
*N/A*	1418320657	10	allergen Fel d 4-like [*Canis lupus dingo*]	0.492	0.222
*ACTN1*	1418347612	5	alpha-actinin-1 isoform X1 [*Canis lupus dingo*]	0.411	0.222
*ACTN1*	1239915208	5	alpha-actinin-1 isoform X2 [*Canis lupus familiaris*]	0.411	0.222
*ACTN1*	1418347616	5	alpha-actinin-1 isoform X3 [*Canis lupus dingo*]	0.411	0.222
*ACTN1*	73963339	5	alpha-actinin-1 isoform X4 [*Canis lupus familiaris*]	0.411	0.222
*ACTN1*	73963357	5	alpha-actinin-1 isoform X5 [*Canis lupus familiaris*]	0.411	0.222
*CP*	1418510222	4	ceruloplasmin isoform X1 [*Canis lupus dingo*]	0.718	0.222
*CP*	928167527	3	ceruloplasmin isoform X1 [*Canis lupus familiaris*]	0.726	0.222
*CP*	1418510226	4	ceruloplasmin isoform X2 [*Canis lupus dingo*]	0.718	0.222
*CP*	1239957836	3	ceruloplasmin isoform X2 [*Canis lupus familiaris*]	0.726	0.222
*CP*	1418510228	4	ceruloplasmin isoform X3 [*Canis lupus dingo*]	0.718	0.222
*CP*	73990367	3	ceruloplasmin isoform X3 [*Canis lupus familiaris*]	0.726	0.222
*CP*	1418510230	4	ceruloplasmin isoform X4 [*Canis lupus dingo*]	0.718	0.222
*CP*	1239957839	3	ceruloplasmin isoform X4 [*Canis lupus familiaris*]	0.726	0.222
*CANF2*	296863542	7	chain A, crystal structure of dog lipocalin allergen Can f 2 and implications for cross-reactivity to the cat allergen Fel d 4	0.538	0.222
*N/A*	1374502923	10	chain D, lipocalin Can f 6 allergen	0.492	0.222
*C3*	1418218430	30	complement C3 [*Canis lupus dingo*]	0.561	0.222
*C3*	1239951704	28	complement C3 [*Canis lupus familiaris*]	0.547	0.222
*FGA*	1304047	2	fibrinogen A-alpha chain, partial [*Canis lupus familiaris*]	0.673	0.222
*G6PD*	1418225081	7	glucose-6-phosphate 1-dehydrogenase isoform X1 [*Canis lupus dingo*]	0.391	0.222
*G6PD*	1418225083	7	glucose-6-phosphate 1-dehydrogenase isoform X2 [*Canis lupus dingo*]	0.391	0.222
*HSP90AB1*	922059102	3	heat shock protein 90 kDa alpha class B member 1, partial [*Canis lupus familiaris*]	0.417	0.222
*IGH-10*	1494245227	5	immunoglobulin heavy chain IGH-10 [*Canis lupus familiaris*]	0.502	0.222
*IGH-12*	1494245231	5	immunoglobulin heavy chain IGH-12 [*Canis lupus familiaris*]	0.502	0.222
*IGH-14*	1494245235	5	immunoglobulin heavy chain IGH-14 [*Canis lupus familiaris*]	0.502	0.222
*IGH-15*	1494245237	5	immunoglobulin heavy chain IGH-15 [*Canis lupus familiaris*]	0.502	0.222
*IGH-16*	1494245239	5	immunoglobulin heavy chain IGH-16 [*Canis lupus familiaris*]	0.502	0.222
*IGH-17*	1494245241	5	immunoglobulin heavy chain IGH-17 [*Canis lupus familiaris*]	0.502	0.222
*IGH-3*	1494245213	5	immunoglobulin heavy chain IGH-3 [*Canis lupus familiaris*]	0.502	0.222
*IGH-7*	1494245221	5	immunoglobulin heavy chain IGH-7 [*Canis lupus familiaris*]	0.502	0.222
*IGH-9*	1494245225	5	immunoglobulin heavy chain IGH-9 [*Canis lupus familiaris*]	0.502	0.222
*KLK1*	1418303494	9	kallikrein-1 isoform X1 [*Canis lupus dingo*]	−0.633	0.222
*KLK1*	1418303496	9	kallikrein-1 isoform X2 [*Canis lupus dingo*]	−0.633	0.222
*KLK1*	55741639	8	kallikrein-1 precursor [*Canis lupus familiaris*]	−0.637	0.222
*KRT13*	1077167933	6	keratin, type I cytoskeletal 13 [*Canis lupus familiaris*]	0.758	0.222
*SERPINB1*	1418205376	11	leukocyte elastase inhibitor [*Canis lupus dingo*]	0.461	0.222
*LDHA*	1418345245	6	L-lactate dehydrogenase A chain-like [*Canis lupus dingo*]	0.429	0.222
*LDHA*	545507351	6	L-lactate dehydrogenase A chain-like [*Canis lupus familiaris*]	0.429	0.222
*DMBT1*	1418246878	11	LOW QUALITY PROTEIN: deleted in malignant brain tumors 1 protein [*Canis lupus dingo*]	−0.419	0.222
*LDHA*	1239958986	5	LOW QUALITY PROTEIN: L-lactate dehydrogenase A chain-like [*Canis lupus familiaris*]	0.647	0.222
*CAPG*	1418211046	4	macrophage-capping protein isoform X2 [*Canis lupus dingo*]	0.453	0.222
*CAPG*	928158112	4	macrophage-capping protein isoform X2 [*Canis lupus familiaris*]	0.453	0.222
*CANF2*	1418266307	7	minor allergen Can f 2 [*Canis lupus dingo*]	0.538	0.222
*MPO*	545511447	10	myeloperoxidase [*Canis lupus familiaris*]	0.546	0.222
*MYH11*	1418286550	5	myosin-11 isoform X1 [*Canis lupus dingo*]	0.470	0.222
*MYH11*	545502107	5	myosin-11 isoform X2 [*Canis lupus familiaris*]	0.470	0.222
*MYH11*	1418286554	5	myosin-11 isoform X3 [*Canis lupus dingo*]	0.470	0.222
*MYH14*	1418286556	5	myosin-11 isoform X4 [*Canis lupus dingo*]	0.470	0.222
*OLFM4*	1239956251	2	olfactomedin-4 [*Canis lupus familiaris*]	−0.529	0.222
*CANF2*	29292272	7	precursor Can f 2, partial [*Canis lupus familiaris*]	0.538	0.222
*GDI2*	1418501820	4	rab GDP dissociation inhibitor beta isoform X1 [*Canis lupus dingo*]	0.275	0.222
*GDI2*	1418501822	4	rab GDP dissociation inhibitor beta isoform X2 [*Canis lupus dingo*]	0.275	0.222
*GDI2*	1418501824	4	rab GDP dissociation inhibitor beta isoform X3 [*Canis lupus dingo*]	0.275	0.222
*CANF2*	3121746	7	Minor allergen Can f 2	0.538	0.222
*SOD1*	1418511817	6	superoxide dismutase [Cu-Zn] [*Canis lupus dingo*]	0.344	0.222
*TXN*	1418321566	4	thioredoxin [*Canis lupus dingo*]	0.346	0.222
*TXN*	1239928271	4	thioredoxin-like isoform X1 [*Canis lupus familiaris*]	0.346	0.222
*TXN*	1239928273	4	thioredoxin-like isoform X2 [*Canis lupus familiaris*]	0.346	0.222
*N/A*	16607663	5	unnamed protein product [*Canis lupus familiaris*]	0.502	0.222
*N/A*	16607675	4	unnamed protein product [*Canis lupus familiaris*]	0.502	0.222
*N/A*	16607718	5	unnamed protein product [*Canis lupus familiaris*]	0.502	0.222
*ANXA2*	1418258110	3	annexin A2 [*Canis lupus dingo*]	0.846	0.229
*HSPA2*	57090217	3	heat shock-related 70 kDa protein 2 [*Canis lupus familiaris*]	0.483	0.229
*KRT2*	1068388058	3	keratin, type II cytoskeletal 2 oral [*Canis lupus familiaris*]	0.841	0.229
*KRT2*	1239965760	3	keratin, type II cytoskeletal 2 oral isoform X1 [*Canis lupus familiaris*]	0.841	0.229
*KRT2*	1418221668	3	keratin, type II cytoskeletal 2 oral-like [*Canis lupus dingo*]	0.841	0.229
*KRT76*	15419605	3	masticatory epithelia keratin 2p [*Canis lupus familiaris*]	0.841	0.229
*NQO2*	1418205354	3	ribosyldihydronicotinamide dehydrogenase [quinone] [*Canis lupus dingo*]	0.502	0.229
*NQO2*	74003808	3	ribosyldihydronicotinamide dehydrogenase [quinone] [*Canis lupus familiaris*]	0.502	0.229
*NQO1*	1418308783	9	NAD(P)H dehydrogenase [quinone] 1 [*Canis lupus dingo*]	0.362	0.254
*NQO1*	545500084	10	NAD(P)H dehydrogenase [quinone] 1 [*Canis lupus familiaris*]	0.362	0.254
*LYZ*	9257149	2	chain A, X-ray crystal structure analysis of canine milk lysozyme (Apo-type)	−0.612	0.286
*LYZ*	13787135	2	chain B, lysozyme C	−0.612	0.286
*LTF*	84872712	8	lactoferrin, partial [*Canis lupus familiaris*]	−0.239	0.286
*CES1*	1418507047	7	liver carboxylesterase 1 [*Canis lupus dingo*]	0.183	0.286
*LDHB*	356461040	4	L-lactate dehydrogenase B chain [*Canis lupus familiaris*]	0.338	0.286
*LDHB*	1418299581	4	L-lactate dehydrogenase B chain-like [*Canis lupus dingo*]	0.338	0.286
*LDHB*	1418327082	3	L-lactate dehydrogenase B chain-like [*Canis lupus dingo*]	0.338	0.286
*LDHB*	1418514557	3	L-lactate dehydrogenase B chain-like [*Canis lupus dingo*]	0.338	0.286
*LYZ*	925114454	2	lysozyme C, milk isozyme-like precursor [*Canis lupus familiaris*]	−0.612	0.286
*NME1*	545510194	2	nucleoside diphosphate kinase A isoform X1 [*Canis lupus familiaris*]	0.560	0.286
*N/A*	124847	4	Double-headed protease inhibitor, submandibular gland	0.596	0.286
*LYZ*	8928188	2	Lysozyme C, milk isozyme;	−0.612	0.286
*YWHAG*	1418307161	4	14-3-3 protein gamma [*Canis lupus dingo*]	0.243	0.310
*YWHAG*	1239905645	4	14-3-3 protein gamma [*Canis lupus familiaris*]	0.243	0.310
*HSPA5*	345806081	5	78 kDa glucose-regulated protein [*Canis lupus familiaris*]	0.372	0.310
*HSPA5*	1418264760	5	endoplasmic reticulum chaperone BiP [*Canis lupus dingo*]	0.372	0.310
*NCCRP1*	73948372	3	F-box only protein 50 [*Canis lupus familiaris*]	0.342	0.310
*FGA*	1418336346	5	fibrinogen-alpha chain [*Canis lupus dingo*]	0.459	0.310
*FGA*	73978329	5	fibrinogen-alpha chain [*Canis lupus familiaris*]	0.459	0.310
*H2AFZ*	559767198	2	H2A histone family, member Z [*Canis lupus familiaris*]	0.417	0.310
*HSP70*	17298186	3	heat shock protein 70 [*Canis lupus familiaris*]	0.451	0.310
*HIST1H2A*	1239979771	2	histone H2A type 1 [*Canis lupus familiaris*]	0.417	0.310
*HIST1H2AA*	57110393	2	histone H2A type 1-A [*Canis lupus familiaris*]	0.417	0.310
*HIST1H2AB*	1418253446	2	histone H2A type 1-B/E [*Canis lupus dingo*]	0.417	0.310
*HIST1H2AC*	545554572	2	histone H2A type 1-C [*Canis lupus familiaris*]	0.417	0.310
*HIST1H2AE*	1418204456	2	histone H2A type 1-E [*Canis lupus dingo*]	0.417	0.310
*HIST1H2AE*	1239979741	2	histone H2A type 1-E [*Canis lupus familiaris*]	0.417	0.310
*HIST1H2AE*	1418253448	2	histone H2A type 1-E-like [*Canis lupus dingo*]	0.417	0.310
*HIST1H2AE*	1418253483	2	histone H2A type 1-E-like [*Canis lupus dingo*]	0.417	0.310
*HIST1H2AE*	74004170	2	histone H2A type 1-E-like [*Canis lupus familiaris*]	0.417	0.310
*HIST1H2AH*	928180938	2	histone H2A type 1-H [*Canis lupus familiaris*]	0.417	0.310
*HIST1H2AH*	1239979774	2	histone H2A type 1-H [*Canis lupus familiaris*]	0.417	0.310
*HIST1H2AH*	1418204540	2	histone H2A type 1-H-like [*Canis lupus dingo*]	0.417	0.310
*HIST2H2AA*	73981516	2	histone H2A type 2-A [*Canis lupus familiaris*]	0.417	0.310
*HIST2H2AC*	359321687	2	histone H2A type 2-C [*Canis lupus familiaris*]	0.417	0.310
*HIST3H2A*	545521932	2	histone H2A type 3 [*Canis lupus familiaris*]	0.417	0.310
*HISTH2AJ*	57106965	2	histone H2A.J [*Canis lupus familiaris*]	0.417	0.310
*HISTH2AV*	928186182	2	histone H2A.V isoform X1 [*Canis lupus familiaris*]	0.417	0.310
*HISTH2AV*	928186186	2	histone H2A.V isoform X3 [*Canis lupus familiaris*]	0.417	0.310
*HISTH2AB*	928180889	2	histone H2A-beta, sperm-like [*Canis lupus familiaris*]	0.417	0.310
*HISTH2AX*	545497152	2	histone H2AX [*Canis lupus familiaris*]	0.417	0.310
*IGJ; JCHAIN*	345779666	6	immunoglobulin J chain [*Canis lupus familiaris*]	−0.278	0.310
*KRT4*	1418221457	9	keratin, type II cytoskeletal 4 [*Canis lupus dingo*]	0.819	0.310
*LPO*	1418338512	22	lactoperoxidase [*Canis lupus dingo*]	−0.840	0.310
*LPO*	1239919186	20	lactoperoxidase [*Canis lupus familiaris*]	−0.810	0.310
*ALB*	1418328547	2	LOW QUALITY PROTEIN: serum albumin-like [*Canis lupus dingo*]	0.302	0.310
*MYL12B*	57089773	2	myosin regulatory light chain 12B [*Canis lupus familiaris*]	0.378	0.310
*MYL9*	1418314603	2	myosin regulatory light polypeptide 9 isoform X1 [*Canis lupus dingo*]	0.378	0.310
*MYL9*	345803346	2	myosin regulatory light polypeptide 9 isoform X2 [*Canis lupus familiaris*]	0.378	0.310
*PGK1*	74007807	4	phosphoglycerate kinase 1 [*Canis lupus familiaris*]	0.232	0.310
*PIP*	73978762	9	prolactin-inducible protein [*Canis lupus familiaris*]	−0.427	0.310
*HSP70*	56749085	3	Heat shock 70 kDa protein 1	0.451	0.310
*SLPI*	164499359	3	secretory leukocyte peptidase inhibitor, partial [*Canis lupus familiaris*]	−0.391	0.310
*TALDO1*	359321944	7	transaldolase [*Canis lupus familiaris*]	0.287	0.310
*ZG16B*	73959451	4	zymogen granule protein 16 homolog B [*Canis lupus familiaris*]	-1.886	0.310
*ACTN2*	1418509128	2	alpha-actinin-2 isoform X1 [*Canis lupus dingo*]	0.250	0.333
*ACTN2*	1418509130	2	alpha-actinin-2 isoform X2 [*Canis lupus dingo*]	0.250	0.333
*ACTN3*	1418503151	2	alpha-actinin-3 isoform X1 [*Canis lupus dingo*]	0.250	0.333
*ACTN3*	1418503153	2	alpha-actinin-3 isoform X2 [*Canis lupus dingo*]	0.250	0.333
*ACTN3*	1418503155	2	alpha-actinin-3 isoform X3 [*Canis lupus dingo*]	0.250	0.333
*GSTO1*	1418245560	3	glutathione S-transferase omega-1 [*Canis lupus dingo*]	0.417	0.333
*UBA1*	928183828	2	LOW QUALITY PROTEIN: ubiquitin-like modifier-activating enzyme 1 [*Canis lupus familiaris*]	0.521	0.333
*ME1*	545519773	2	NADP-dependent malic enzyme [*Canis lupus familiaris*]	0.802	0.333
*ME1*	1418348746	2	NADP-dependent malic enzyme isoform X1 [*Canis lupus dingo*]	0.802	0.333
*ME1*	1418348750	2	NADP-dependent malic enzyme isoform X3 [*Canis lupus dingo*]	0.802	0.333
*UBA1*	1418249227	2	ubiquitin-like modifier-activating enzyme 1 [*Canis lupus dingo*]	0.521	0.333
*ARPC5*	1418316200	2	actin-related protein 2/3 complex subunit 5 [*Canis lupus dingo*]	0.687	0.400
*ADIRF*	1239898218	2	adipogenesis regulatory factor [*Canis lupus familiaris*]	−0.128	0.400
*CASP14*	1239951047	2	LOW QUALITY PROTEIN: caspase-14 [*Canis lupus familiaris*]	0.558	0.400
*MUC5B*	1239945861	2	mucin-5B isoform X1 [*Canis lupus familiaris*]	0.500	0.400
*UPP1*	1418511026	3	uridine phosphorylase 1 [*Canis lupus dingo*]	0.851	0.400
*UPP1*	1239883228	3	uridine phosphorylase 1-like [*Canis lupus familiaris*]	0.851	0.400
*PPIA*	1418214537	2	ovostatin homolog 2-like [*Canis lupus dingo*]	0.035	0.413
*YWHAS*	1418505824	11	14-3-3 protein sigma [*Canis lupus dingo*]	0.311	0.421
*ACT*	1418333042	2	actin, clone 302-like [*Canis lupus dingo*]	−0.298	0.421
*CA2*	223556019	2	carbonic anhydrase 2 [*Canis lupus familiaris*]	0.096	0.421
*CAMP*	1418215404	6	cathelicidin antimicrobial peptide [*Canis lupus dingo*]	0.290	0.421
*CAMP*	50979214	6	cathelicidin antimicrobial peptide precursor [*Canis lupus familiaris*]	0.290	0.421
*CRISP2*	545519428	6	cysteine-rich secretory protein 2 isoform X1 [*Canis lupus familiaris*]	−0.357	0.421
*CRISP2*	1239929902	6	cysteine-rich secretory protein 2 isoform X2 [*Canis lupus familiaris*]	−0.357	0.421
*FGB*	1418336465	4	fibrinogen beta chain [*Canis lupus dingo*]	0.473	0.421
*MMP9*	2564101	7	gelatinase B [*Canis lupus familiaris*]	0.351	0.421
*GDA*	1418301814	4	guanine deaminase [*Canis lupus dingo*]	0.474	0.421
*FCGBP*	1418305257	2	IgGFc-binding protein isoform X1 [*Canis lupus dingo*]	−1.045	0.421
*FCGBP*	1418305259	2	IgGFc-binding protein isoform X2 [*Canis lupus dingo*]	−1.004	0.421
*IGH-CH4*	124390013	2	immunoglobulin heavy chain constant region CH4, partial [*Canis lupus familiaris*]	−0.255	0.421
*IGHJ*	1340239380	2	immunoglobulin heavy chain variable region, partial [*Canis lupus familiaris*]	−0.692	0.421
*IGM*	146743249	2	immunoglobulin mu heavy chain variable region, partial [*Canis lupus familiaris*]	−0.255	0.421
*KRT1*	1418221463	4	keratin, type II cytoskeletal 1 [*Canis lupus dingo*]	0.583	0.421
*KRT5*	1418221469	4	keratin, type II cytoskeletal 5 [*Canis lupus dingo*]	0.693	0.421
*KRT5*	1069415302	4	keratin, type II cytoskeletal 5 [*Canis lupus familiaris*]	0.693	0.421
*LTF*	339305353	2	lactotransferrin, partial [*Canis lupus familiaris*]	−0.424	0.421
*GLO1*	345778725	2	lactoylglutathione lyase [*Canis lupus familiaris*]	0.184	0.421
*GPI*	1418305580	15	LOW QUALITY PROTEIN: glucose-6-phosphate isomerase [*Canis lupus dingo*]	0.240	0.421
*MMP9*	1418252189	7	matrix metalloproteinase-9 [*Canis lupus dingo*]	0.351	0.421
*MMP9*	11034716	7	matrix metalloproteinase-9 [*Canis lupus familiaris*]	0.351	0.421
*MMP9*	50978992	6	matrix metalloproteinase-9 precursor [*Canis lupus familiaris*]	0.363	0.421
*MYL6*	345776590	3	myosin light polypeptide 6 isoform X1 [*Canis lupus familiaris*]	0.382	0.421
*MYL6*	1418269617	3	myosin light polypeptide 6 isoform X2 [*Canis lupus dingo*]	0.382	0.421
*MYL6*	1239922056	3	myosin light polypeptide 6 isoform X3 [*Canis lupus familiaris*]	0.382	0.421
*SLPI*	158936956	4	protease inhibitor [*Canis lupus familiaris*]	−0.439	0.421
*KRT1*	75062693	4	Keratin, type II cytoskeletal 1;	0.583	0.421
*TPM3*	1418313468	2	tropomyosin alpha-3 chain isoform X7 [*Canis lupus dingo*]	0.694	0.421
*MUC19*	1239968164	7	mucin-19 [*Canis lupus familiaris*]	0.020	0.486
*SERPINA1*	119637732	6	alpha-1 antitrypsin [*Canis lupus familiaris*]	−0.547	0.548
*SERPINA1*	1239913623	6	alpha-1-antitrypsin isoform X1 [*Canis lupus familiaris*]	−0.547	0.548
*SERPINA1*	1418345511	6	alpha-1-antitrypsin-like [*Canis lupus dingo*]	−0.547	0.548
*A2M*	1418213974	3	alpha-2-macroglobulin [*Canis lupus dingo*]	0.389	0.548
*A2M*	345792424	3	alpha-2-macroglobulin [*Canis lupus familiaris*]	0.389	0.548
*ANGPTL5*	1418194692	6	angiopoietin-related protein 5-like [*Canis lupus dingo*]	−0.465	0.548
*LOC611632*	1418510295	8	ceruloplasmin-like [*Canis lupus dingo*]	0.668	0.548
*LOC611632*	345788999	7	ceruloplasmin-like [*Canis lupus familiaris*]	0.668	0.548
*CFL1*	57099669	6	cofilin-1 [*Canis lupus familiaris*]	0.000	0.548
*CFL2*	1418315363	5	cofilin-1-like [*Canis lupus dingo*]	−0.005	0.548
*SOD1*	18150346	5	Cu/Zn superoxide dismutase [*Canis lupus familiaris*]	0.214	0.548
*DMBT1*	928175938	12	deleted in malignant brain tumors 1 protein isoform X1 [*Canis lupus familiaris*]	−0.364	0.548
*DMBT1*	928175949	12	deleted in malignant brain tumors 1 protein isoform X2 [*Canis lupus familiaris*]	−0.364	0.548
*DMBT1*	928175953	12	deleted in malignant brain tumors 1 protein isoform X3 [*Canis lupus familiaris*]	−0.364	0.548
*FABP5*	928174762	4	fatty acid-binding protein, epidermal [*Canis lupus familiaris*]	0.339	0.548
*FETUB*	1418515534	2	fetuin-B [*Canis lupus dingo*]	−0.083	0.548
*FETUB*	74003556	3	fetuin-B [*Canis lupus familiaris*]	−0.021	0.548
*PYGL*	562155348	13	glycogen phosphorylase, liver form [*Canis lupus familiaris*]	0.255	0.548
*IGH-4*	1494245215	6	immunoglobulin heavy chain IGH-4 [*Canis lupus familiaris*]	0.351	0.548
*SERPINB1*	1239979297	6	leukocyte elastase inhibitor [*Canis lupus familiaris*]	0.234	0.548
*MSN*	545558304	6	moesin isoform X1 [*Canis lupus familiaris*]	0.309	0.548
*MSN*	1418243364	6	moesin isoform X2 [*Canis lupus dingo*]	0.309	0.548
*MSN*	928185024	6	moesin isoform X3 [*Canis lupus familiaris*]	0.309	0.548
*OPRPN*	1418328391	8	opiorphin prepropeptide [*Canis lupus dingo*]	0.082	0.548
*TGM3*	1418250613	11	protein-glutamine gamma-glutamyltransferase E [*Canis lupus dingo*]	0.273	0.548
*PKM*	1239972768	7	pyruvate kinase PKM isoform X1 [*Canis lupus familiaris*]	−0.157	0.548
*PKM*	1418257040	6	pyruvate kinase PKM isoform X2 [*Canis lupus dingo*]	−0.168	0.548
*PKM*	1418257042	7	pyruvate kinase PKM isoform X3 [*Canis lupus dingo*]	−0.157	0.548
*PKM*	545550333	6	pyruvate kinase PKM isoform X3 [*Canis lupus familiaris*]	−0.168	0.548
*PKM*	1418257044	6	pyruvate kinase PKM isoform X4 [*Canis lupus dingo*]	−0.130	0.548
*MMP9*	4868451	3	type IV collagenase MMP-9, partial [*Canis lupus familiaris*]	0.344	0.548
*N/A*	16607721	6	unnamed protein product [*Canis lupus familiaris*]	0.351	0.548
*HSP90AA1*	734548054	2	heat shock protein HSP 90-alpha [*Toxocara canis*]	0.194	0.556
*HYAL1*	1239950142	7	hyaluronidase-1 isoform X3 [*Canis lupus familiaris*]	0.404	0.556
*IGH-CH2*	124390009	2	immunoglobulin heavy chain constant region CH2, partial [*Canis lupus familiaris*]	0.308	0.556
*SCGB2A2*	928186528	2	mammaglobin-A-like [*Canis lupus familiaris*]	1.489	0.556
*PADI4*	1418507405	2	protein-arginine deiminase type-4 [*Canis lupus dingo*]	0.388	0.556
*N/A*	1535091294	2	unnamed protein product [*Toxocara canis*]	0.194	0.556
*MYH10*	1418312500	4	myosin-10 isoform X2 [*Canis lupus dingo*]	0.405	0.571
*MYH10*	928134337	4	myosin-10 isoform X2 [*Canis lupus familiaris*]	0.405	0.571
*MYH10*	928134341	4	myosin-10 isoform X4 [*Canis lupus familiaris*]	0.405	0.571
*MYH10*	1418312506	4	myosin-10 isoform X5 [*Canis lupus dingo*]	0.405	0.571
*MYH10*	928134343	4	myosin-10 isoform X5 [*Canis lupus familiaris*]	0.405	0.571
*MYH10*	1418312512	4	myosin-10 isoform X6 [*Canis lupus dingo*]	0.405	0.571
*C4A*	1239928570	6	complement C4-A [*Canis lupus familiaris*]	0.202	0.629
*C4A*	1418290456	6	complement C4-A-like [*Canis lupus dingo*]	0.202	0.629
*KNG1*	1418515459	3	kininogen-1 isoform X1 [*Canis lupus dingo*]	0.070	0.629
*KNG1*	345796419	3	kininogen-1 isoform X1 [*Canis lupus familiaris*]	0.070	0.629
*KNG1*	1418515461	3	kininogen-1 isoform X2 [*Canis lupus dingo*]	0.070	0.629
*KNG1*	57109938	3	kininogen-1 isoform X2 [*Canis lupus familiaris*]	0.070	0.629
*LDHA*	1239955652	3	L-lactate dehydrogenase A chain-like isoform X1 [*Canis lupus familiaris*]	0.430	0.629
*PGM1*	928134704	2	phosphoglucomutase-1 isoform X1 [*Canis lupus familiaris*]	0.330	0.629
*PGM1*	73956158	2	phosphoglucomutase-1 isoform X2 [*Canis lupus familiaris*]	0.330	0.629
*LOC611458*	1418213980	5	pregnancy zone protein-like isoform X3 [*Canis lupus dingo*]	0.180	0.629
*LOC611458*	1239967247	5	pregnancy zone protein-like isoform X3 [*Canis lupus familiaris*]	0.180	0.629
*TUBA1B*	1418342112	2	tubulin alpha-1B chain isoform X1 [*Canis lupus dingo*]	0.544	0.629
*TUBA1B*	1418342114	2	tubulin alpha-1B chain isoform X2 [*Canis lupus dingo*]	0.544	0.629
*TUBA8*	1418342116	2	tubulin alpha-8 chain isoform X3 [*Canis lupus dingo*]	0.544	0.629
*LOC112676265*	1418260227	2	uncharacterized protein LOC112676265 [*Canis lupus dingo*]	−0.704	0.629
*RPLP2*	1418207290	2	60S acidic ribosomal protein P2 [*Canis lupus dingo*]	0.445	0.667
*A2ML1*	1418213935	6	alpha-2-macroglobulin-like protein 1 isoform X1 [*Canis lupus dingo*]	0.561	0.667
*A2ML1*	1239967618	6	alpha-2-macroglobulin-like protein 1 isoform X1 [*Canis lupus familiaris*]	0.561	0.667
*A2ML1*	1418213939	6	alpha-2-macroglobulin-like protein 1 isoform X2 [*Canis lupus dingo*]	0.561	0.667
*A2ML1*	1239967622	6	alpha-2-macroglobulin-like protein 1 isoform X2 [*Canis lupus familiaris*]	0.561	0.667
*BPIFA1*	73992235	2	BPI fold-containing family A member 1 [*Canis lupus familiaris*]	0.471	0.667
*CAT*	4115557	2	catalase [*Canis lupus familiaris*]	−0.006	0.667
*CAT*	12082093	2	catalase [*Canis lupus familiaris*]	−0.006	0.667
*CAPZB*	928128985	2	F-actin-capping protein subunit beta isoform X1 [*Canis lupus familiaris*]	1.088	0.667
*CAPZB*	928128987	3	F-actin-capping protein subunit beta isoform X2 [*Canis lupus familiaris*]	1.088	0.667
*CAPZB*	1418507824	2	F-actin-capping protein subunit beta isoform X3 [*Canis lupus dingo*]	1.088	0.667
*CAPZB*	1239893342	2	F-actin-capping protein subunit beta isoform X3 [*Canis lupus familiaris*]	1.088	0.667
*CAPZB*	545492085	3	F-actin-capping protein subunit beta isoform X4 [*Canis lupus familiaris*]	1.088	0.667
*CAPZB*	1239893345	2	F-actin-capping protein subunit beta isoform X5 [*Canis lupus familiaris*]	1.088	0.667
*CAPZB*	1239893349	2	F-actin-capping protein subunit beta isoform X7 [*Canis lupus familiaris*]	1.088	0.667
*FOLR2*	1418222123	2	folate receptor beta isoform X1 [*Canis lupus dingo*]	1.376	0.667
*FOLR2*	1239953679	2	folate receptor beta isoform X1 [*Canis lupus familiaris*]	1.376	0.667
*FOLR2*	1418222127	2	folate receptor beta isoform X2 [*Canis lupus dingo*]	1.376	0.667
*FOLR2*	928165121	2	folate receptor beta isoform X2 [*Canis lupus familiaris*]	1.376	0.667
*HBE*	1418222280	2	hemoglobin subunit epsilon [*Canis lupus dingo*]	−0.898	0.667
*KRT14*	1418338716	3	keratin, type I cytoskeletal 14 [*Canis lupus dingo*]	0.845	0.667
*KRT24*	1418339572	3	keratin, type I cytoskeletal 24 [*Canis lupus dingo*]	1.420	0.667
*KRT24*	345805399	3	keratin, type I cytoskeletal 24 [*Canis lupus familiaris*]	1.420	0.667
*KRT78*	73996461	2	keratin, type II cytoskeletal 78 [*Canis lupus familiaris*]	2.219	0.667
*MGAM*	1418324508	2	maltase-glucoamylase, intestinal isoform X1 [*Canis lupus dingo*]	0.923	0.667
*MGAM*	1239938912	2	maltase-glucoamylase, intestinal isoform X1 [*Canis lupus familiaris*]	0.923	0.667
*MGAM*	1418324514	2	maltase-glucoamylase, intestinal isoform X2 [*Canis lupus dingo*]	0.923	0.667
*MGAM*	928156540	2	maltase-glucoamylase, intestinal isoform X2 [*Canis lupus familiaris*]	0.923	0.667
*MUC4*	1418200086	3	mucin-4 [*Canis lupus dingo*]	0.994	0.667
*MUC4*	1239976879	3	mucin-4 [*Canis lupus familiaris*]	0.994	0.667
*PLG*	558695388	2	plasminogen precursor [*Canis lupus familiaris*]	0.726	0.667
*PSMA1*	1418204605	3	proteasome subunit alpha type-1 [*Canis lupus dingo*]	0.708	0.667
*PSMA1*	1418222965	3	proteasome subunit alpha type-1 [*Canis lupus dingo*]	0.708	0.667
*PSMA1*	1418258143	2	proteasome subunit alpha type-1-like [*Canis lupus dingo*]	1.060	0.667
*PSMA3*	1418345461	3	proteasome subunit alpha type-3 [*Canis lupus dingo*]	1.737	0.667
*PSMA3*	73963050	3	proteasome subunit alpha type-3 [*Canis lupus familiaris*]	1.737	0.667
*PSMA4*	1418514065	2	proteasome subunit alpha type-4 [*Canis lupus dingo*]	0.829	0.667
*OXCT1*	1418329836	2	succinyl-CoA:3-ketoacid coenzyme A transferase 1, mitochondrial [*Canis lupus dingo*]	0.631	0.667
*OXCT1*	928131428	2	succinyl-CoA:3-ketoacid coenzyme A transferase 1, mitochondrial [*Canis lupus familiaris*]	0.631	0.667
*WDR1*	195546936	3	WD repeat-containing protein 1 [*Canis lupus familiaris*]	0.712	0.667
*GSN*	1239925762	7	gelsolin [*Canis lupus familiaris*]	−0.278	0.675
*GSN*	1418322411	7	gelsolin isoform X1 [*Canis lupus dingo*]	−0.278	0.675
*GSN*	1418322413	7	gelsolin isoform X2 [*Canis lupus dingo*]	−0.278	0.675
*GSN*	1418322415	7	gelsolin isoform X3 [*Canis lupus dingo*]	−0.278	0.675
*YWHAB*	1418252030	3	14-3-3 protein beta/alpha [*Canis lupus dingo*]	0.252	0.690
*ACTB*	5597005	2	beta-actin [*Canis lupus familiaris*]	−0.175	0.690
*MPO*	494395	3	chain C, myeloperoxidase	0.360	0.690
*COTL1*	1418309542	3	coactosin-like protein [*Canis lupus dingo*]	0.318	0.690
*COTL1*	359319594	3	coactosin-like protein, partial [*Canis lupus familiaris*]	0.318	0.690
*FABP5*	1418323952	3	fatty acid-binding protein 5 [*Canis lupus dingo*]	0.227	0.690
*FABP5*	1239986810	3	fatty acid-binding protein, epidermal [*Canis lupus familiaris*]	0.227	0.690
*FABP5*	1239971228	3	fatty acid-binding protein, epidermal isoform X1 [*Canis lupus familiaris*]	0.227	0.690
*FABP5*	1239971230	3	fatty acid-binding protein, epidermal isoform X2 [*Canis lupus familiaris*]	0.227	0.690
*FABP5*	1239971232	3	fatty acid-binding protein, epidermal isoform X3 [*Canis lupus familiaris*]	0.227	0.690
*IGH-1*	1494245209	7	immunoglobulin heavy chain IGH-1 [*Canis lupus familiaris*]	0.285	0.690
*IGH-11*	1494245229	7	immunoglobulin heavy chain IGH-11 [*Canis lupus familiaris*]	0.312	0.690
*IGH-13*	1494245233	7	immunoglobulin heavy chain IGH-13 [*Canis lupus familiaris*]	0.312	0.690
*SCGB2A2*	359321930	3	mammaglobin-A isoform X2 [*Canis lupus familiaris*]	0.143	0.690
*SCGB2A1*	1239883349	4	mammaglobin-B-like isoform X2 [*Canis lupus familiaris*]	0.143	0.690
*PRDX6*	1418316704	3	peroxiredoxin-6 [*Canis lupus dingo*]	0.289	0.690
*PRDX6*	1239911065	3	peroxiredoxin-6 [*Canis lupus familiaris*]	0.289	0.690
*PFN1*	1418313130	5	profilin-1 [*Canis lupus dingo*]	0.005	0.690
*PFN1*	1239901610	3	profilin-1 [*Canis lupus familiaris*]	0.061	0.690
*VDB*	1418328657	8	vitamin D-binding protein [*Canis lupus dingo*]	0.222	0.690
*ORM1*	1418322788	2	alpha-1-acid glycoprotein 1-like [*Canis lupus dingo*]	0.461	0.700
*SLPI*	1418249573	2	antileukoproteinase-like [*Canis lupus dingo*]	−0.016	0.700
*EFHD2*	1418506694	2	EF-hand domain-containing protein D2 [*Canis lupus dingo*]	0.355	0.700
*EFHD2*	928129121	2	EF-hand domain-containing protein D2 [*Canis lupus familiaris*]	0.355	0.700
*GSTA2*	1418292147	2	glutathione S-transferase A2 [*Canis lupus dingo*]	0.276	0.700
*GSTA3*	635545472	2	glutathione S-transferase alpha 3 [*Canis lupus familiaris*]	0.276	0.700
*GSTA3*	649656024	2	glutathione s-transferase alpha 3 [*Canis lupus familiaris*]	0.276	0.700
*IGH*	208342038	2	immunoglobulin heavy chain variable region, partial [*Canis lupus familiaris*]	0.059	0.700
*KRT6A*	545545386	6	keratin, type II cytoskeletal 6A isoform X1 [*Canis lupus familiaris*]	0.264	0.700
*KRT6A*	345791839	6	keratin, type II cytoskeletal 6A isoform X2 [*Canis lupus familiaris*]	0.264	0.700
*KRT6A*	1418221475	6	keratin, type II cytoskeletal 6A-like [*Canis lupus dingo*]	0.264	0.700
*PLA2G7*	1585652	2	platelet-activating factor acetylhydrolase	−0.337	0.700
*PLA2G7*	1418291961	2	platelet-activating factor acetylhydrolase isoform X1 [*Canis lupus dingo*]	−0.337	0.700
*PLA2G7*	545518207	2	platelet-activating factor acetylhydrolase isoform X1 [*Canis lupus familiaris*]	−0.337	0.700
*PLA2G7*	545518209	2	platelet-activating factor acetylhydrolase isoform X2 [*Canis lupus familiaris*]	−0.337	0.700
*PLA2G7*	1418291973	2	platelet-activating factor acetylhydrolase isoform X3 [*Canis lupus dingo*]	−0.337	0.700
*AZGP1*	70909945	4	zinc alpha-2-glycoprotein 1, partial [*Canis lupus familiaris*]	−0.295	0.700
*AZGP1*	560879429	4	zinc-alpha-2-glycoprotein precursor [*Canis lupus familiaris*]	−0.295	0.700
*ACT4*	734547721	2	actin-4 [*Toxocara canis*]	−0.101	0.730
*ALB*	6687188	2	albumin [*Canis lupus familiaris*]	−0.637	0.730
*AHSG*	1418515495	2	alpha-2-HS-glycoprotein [*Canis lupus dingo*]	−0.685	0.730
*CALR*	1418219369	6	calreticulin [*Canis lupus dingo*]	0.306	0.730
*CALR*	345787749	6	calreticulin [*Canis lupus familiaris*]	0.306	0.730
*ALB*	1104685307	2	chain A, serum albumin	−0.637	0.730
*PPIA*	8699209	4	cyclophilin A, partial [*Canis lupus familiaris*]	0.099	0.730
*GSTM3*	57088159	4	glutathione S-transferase Mu 3 isoform X2 [*Canis lupus familiaris*]	−0.020	0.730
*IVL*	1418213542	5	involucrin [*Canis lupus dingo*]	−0.226	0.730
*IVL*	545528998	5	involucrin [*Canis lupus familiaris*]	−0.226	0.730
*LTF*	339305351	2	lactotransferrin, partial [*Canis lupus familiaris*]	−0.021	0.730
*LDHB*	1418273448	3	L-lactate dehydrogenase B chain-like [*Canis lupus dingo*]	0.110	0.730
*LDHB*	1418319535	3	L-lactate dehydrogenase B chain-like [*Canis lupus dingo*]	0.110	0.730
*PPIA*	1418202784	4	peptidyl-prolyl cis-trans isomerase A isoform X1 [*Canis lupus dingo*]	0.099	0.730
*IVL*	124728	5	Involucrin	−0.226	0.730
*TFF3*	1418228196	2	trefoil factor 3 [*Canis lupus dingo*]	−0.095	0.730
*TFF3*	156875888	2	trefoil factor family peptide 3 [*Canis lupus familiaris*]	−0.095	0.730
*TFF3*	157061758	2	trefoil factor family peptide 3, partial [*Canis lupus*]	−0.095	0.730
*TPM3*	545504091	2	tropomyosin alpha-3 chain isoform X6 [*Canis lupus familiaris*]	0.444	0.730
*A1BG*	1418297237	3	alpha-1B-glycoprotein [*Canis lupus dingo*]	−0.109	0.841
*A1BG*	545487024	3	alpha-1B-glycoprotein [*Canis lupus familiaris*]	−0.109	0.841
*BPIFB1*	1418251074	13	BPI fold-containing family B member 1 [*Canis lupus dingo*]	−1.300	0.841
*BPIFB1*	545540364	15	BPI fold-containing family B member 1 isoform X1 [*Canis lupus familiaris*]	−1.327	0.841
*BPIFB1*	57104142	15	BPI fold-containing family B member 1 isoform X2 [*Canis lupus familiaris*]	−1.327	0.841
*DSC2*	3413469	6	desmocollin type 2, partial [*Canis lupus familiaris*]	−0.098	0.841
*DSC2*	1418314263	6	desmocollin-2 isoform X2 [*Canis lupus dingo*]	−0.098	0.841
*DSC2*	545506022	6	desmocollin-2 isoform X2 [*Canis lupus familiaris*]	−0.098	0.841
*DSC2*	1418314267	6	desmocollin-2 isoform X4 [*Canis lupus dingo*]	−0.098	0.841
*DSC2*	545506026	6	desmocollin-2 isoform X4 [*Canis lupus familiaris*]	−0.098	0.841
*ENO2*	1418256156	2	gamma-enolase [*Canis lupus dingo*]	0.256	0.841
*GAPDH*	6983847	2	glyceraldehyde-3-phosphate dehydrogenase [*Canis lupus familiaris*]	0.041	0.841
*GAPDH*	925115133	2	glyceraldehyde-3-phosphate dehydrogenase [*Canis lupus familiaris*]	0.041	0.841
*GAPDH*	1418292394	2	glyceraldehyde-3-phosphate dehydrogenase isoform X1 [*Canis lupus dingo*]	0.041	0.841
*GAPDH*	1418292396	2	glyceraldehyde-3-phosphate dehydrogenase isoform X2 [*Canis lupus dingo*]	0.041	0.841
*GAPDH*	1418260233	2	glyceraldehyde-3-phosphate dehydrogenase-like [*Canis lupus dingo*]	0.041	0.841
*GAPDH*	1239963916	2	glyceraldehyde-3-phosphate dehydrogenase-like [*Canis lupus familiaris*]	0.041	0.841
*GAPDH*	1418243839	2	glyceraldehyde-3-phosphate dehydrogenase-like isoform X1 [*Canis lupus dingo*]	0.041	0.841
*GAPDH*	1418243841	2	glyceraldehyde-3-phosphate dehydrogenase-like isoform X2 [*Canis lupus dingo*]	0.041	0.841
*CLU*	163954	8	glycoprotein 80 [*Canis lupus familiaris*]	0.132	0.841
*IGHAC*	598107	17	IgA heavy chain constant region, partial [*Canis lupus familiaris*]	−0.223	0.841
*IGHJ*	1340189905	2	immunoglobulin heavy chain variable region, partial [*Canis lupus familiaris*]	−0.803	0.841
*IGL-1*	1494245195	2	immunoglobulin light chain IGL-1, partial [*Canis lupus familiaris*]	0.157	0.841
*IGL-3*	1494245199	2	immunoglobulin light chain IGL-3, partial [*Canis lupus familiaris*]	0.157	0.841
*IGL-7*	1494245207	2	immunoglobulin light chain IGL-7, partial [*Canis lupus familiaris*]	0.157	0.841
*LOC106559694*	928153482	2	LOW QUALITY PROTEIN: glyceraldehyde-3-phosphate dehydrogenase-like [*Canis lupus familiaris*]	0.041	0.841
*MUC7*	1239933320	2	mucin-7 [*Canis lupus familiaris*]	−0.074	0.841
*CANF2*	29292274	5	precursor Can f 2, partial [*Canis lupus familiaris*]	0.318	0.841
*PDIA3*	73964749	7	protein disulfide-isomerase [*Canis lupus familiaris*]	0.037	0.841
*SMR3A*	1239933313	6	submaxillary gland androgen-regulated protein 3A isoform X1 [*Canis lupus familiaris*]	0.075	0.841
*SMR3A*	345779658	6	submaxillary gland androgen-regulated protein 3A isoform X2 [*Canis lupus familiaris*]	0.075	0.841
*N/A*	60734606	6	unnamed protein product, partial [*Canis lupus familiaris*]	−0.047	0.841
*N/A*	60734607	8	unnamed protein product, partial [*Canis lupus familiaris*]	−0.272	0.841
*PYGL*	7595920	2	glycogen phosphorylase, partial [*Canis lupus familiaris*]	0.071	0.857
*LOC611458*	1418213976	6	pregnancy zone protein-like isoform X1 [*Canis lupus dingo*]	0.161	0.857
*LOC611458*	545546412	6	pregnancy zone protein-like isoform X1 [*Canis lupus familiaris*]	0.161	0.857
*LOC611458*	1418213978	6	pregnancy zone protein-like isoform X2 [*Canis lupus dingo*]	0.161	0.857
*LOC611458*	545546414	6	pregnancy zone protein-like isoform X2 [*Canis lupus familiaris*]	0.161	0.857
*GDI2*	50978926	4	rab GDP dissociation inhibitor beta [*Canis lupus familiaris*]	−0.102	0.857
*SERPINB5*	1418298970	5	serpin B5 isoform X1 [*Canis lupus dingo*]	0.288	0.857
*SERPINB5*	345784333	5	serpin B5 isoform X1 [*Canis lupus familiaris*]	0.288	0.857
*SERPINB5*	1418298974	5	serpin B5 isoform X2 [*Canis lupus dingo*]	0.288	0.857
*SERPINB5*	1239884495	5	serpin B5 isoform X2 [*Canis lupus familiaris*]	0.288	0.857
*TAGLN2*	345797882	3	transgelin-2 [*Canis lupus familiaris*]	0.237	0.857
*VIM*	559098393	2	vimentin [*Canis lupus familiaris*]	0.136	0.857
*HBB*	1418222276	7	hemoglobin subunit beta-like [*Canis lupus dingo*]	0.121	0.886
*0*	1418192575	7	submaxillary mucin-like protein, partial [*Canis lupus dingo*]	−0.014	0.886
*HBA1*	1101972892	9	TPA: globin A1 [*Canis lupus familiaris*]	0.103	0.886
*BPIFB2*	1418249549	11	BPI fold-containing family B member 2 [*Canis lupus dingo*]	0.099	0.905
*PGAM1*	345792633	3	phosphoglycerate mutase 1 [*Canis lupus familiaris*]	−0.095	0.905
*LTF*	1418220429	33	lactotransferrin [*Canis lupus dingo*]	0.073	0.937
*PGD*	1418514978	10	6-phosphogluconate dehydrogenase, decarboxylating [*Canis lupus dingo*]	0.100	1.000
*PYCARD*	928137006	2	apoptosis-associated speck-like protein containing a CARD isoform X1 [*Canis lupus familiaris*]	−0.074	1.000
*PYCARD*	1418282931	2	apoptosis-associated speck-like protein containing a CARD isoform X2 [*Canis lupus dingo*]	−0.074	1.000
*ACTB*	1222669	2	beta-actin, partial [*Canis lupus familiaris*]	−0.040	1.000
*BPIFA2*	1418249553	16	BPI fold-containing family A member 2 [*Canis lupus dingo*]	−1.143	1.000
*CAST*	1418331380	2	calpastatin isoform X1 [*Canis lupus dingo*]	0.353	1.000
*CAST*	545492660	2	calpastatin isoform X1 [*Canis lupus familiaris*]	0.353	1.000
*CAST*	1418331398	2	calpastatin isoform X10 [*Canis lupus dingo*]	0.353	1.000
*CAST*	928129400	2	calpastatin isoform X10 [*Canis lupus familiaris*]	0.353	1.000
*CAST*	1418331400	2	calpastatin isoform X11 [*Canis lupus dingo*]	0.353	1.000
*CAST*	928129402	2	calpastatin isoform X11 [*Canis lupus familiaris*]	0.353	1.000
*CAST*	1418331402	2	calpastatin isoform X12 [*Canis lupus dingo*]	0.353	1.000
*CAST*	928129404	2	calpastatin isoform X12 [*Canis lupus familiaris*]	0.353	1.000
*CAST*	1418331404	2	calpastatin isoform X13 [*Canis lupus dingo*]	0.353	1.000
*CAST*	1239894403	2	calpastatin isoform X13 [*Canis lupus familiaris*]	0.353	1.000
*CAST*	1418331406	2	calpastatin isoform X14 [*Canis lupus dingo*]	0.353	1.000
*CAST*	928129406	2	calpastatin isoform X14 [*Canis lupus familiaris*]	0.353	1.000
*CAST*	1418331408	2	calpastatin isoform X15 [*Canis lupus dingo*]	0.353	1.000
*CAST*	928129408	2	calpastatin isoform X15 [*Canis lupus familiaris*]	0.353	1.000
*CAST*	1418331410	2	calpastatin isoform X16 [*Canis lupus dingo*]	0.353	1.000
*CAST*	928129410	2	calpastatin isoform X16 [*Canis lupus familiaris*]	0.353	1.000
*CAST*	1418331412	2	calpastatin isoform X17 [*Canis lupus dingo*]	0.353	1.000
*CAST*	928129416	2	calpastatin isoform X17 [*Canis lupus familiaris*]	0.353	1.000
*CAST*	1418331414	2	calpastatin isoform X18 [*Canis lupus dingo*]	0.353	1.000
*CAST*	928129412	2	calpastatin isoform X18 [*Canis lupus familiaris*]	0.353	1.000
*CAST*	1418331416	2	calpastatin isoform X19 [*Canis lupus dingo*]	0.353	1.000
*CAST*	928129414	2	calpastatin isoform X19 [*Canis lupus familiaris*]	0.353	1.000
*CAST*	1418331382	2	calpastatin isoform X2 [*Canis lupus dingo*]	0.353	1.000
*CAST*	928129389	2	calpastatin isoform X2 [*Canis lupus familiaris*]	0.353	1.000
*CAST*	1239894411	2	calpastatin isoform X20 [*Canis lupus familiaris*]	0.353	1.000
*CAST*	1418331384	2	calpastatin isoform X3 [*Canis lupus dingo*]	0.353	1.000
*CAST*	928129391	2	calpastatin isoform X3 [*Canis lupus familiaris*]	0.353	1.000
*CAST*	1418331386	2	calpastatin isoform X4 [*Canis lupus dingo*]	0.353	1.000
*CAST*	545492662	2	calpastatin isoform X4 [*Canis lupus familiaris*]	0.353	1.000
*CAST*	1418331388	2	calpastatin isoform X5 [*Canis lupus dingo*]	0.353	1.000
*CAST*	928129394	2	calpastatin isoform X5 [*Canis lupus familiaris*]	0.353	1.000
*CAST*	1418331390	2	calpastatin isoform X6 [*Canis lupus dingo*]	0.353	1.000
*CAST*	928129396	2	calpastatin isoform X6 [*Canis lupus familiaris*]	0.353	1.000
*CAST*	1418331392	2	calpastatin isoform X7 [*Canis lupus dingo*]	0.353	1.000
*CAST*	1239894395	2	calpastatin isoform X7 [*Canis lupus familiaris*]	0.353	1.000
*CAST*	1418331394	2	calpastatin isoform X8 [*Canis lupus dingo*]	0.353	1.000
*CAST*	1239894397	2	calpastatin isoform X8 [*Canis lupus familiaris*]	0.353	1.000
*CAST*	1418331396	2	calpastatin isoform X9 [*Canis lupus dingo*]	0.353	1.000
*CAST*	928129398	2	calpastatin isoform X9 [*Canis lupus familiaris*]	0.353	1.000
*CTSG*	928141642	2	cathepsin G [*Canis lupus familiaris*]	0.229	1.000
*HSP90B1*	39654740	2	chain A, endoplasmin	0.074	1.000
*HSP90B1*	71042092	2	chain A, endoplasmin	0.074	1.000
*HSP90B1*	116667120	2	chain A, endoplasmin	0.074	1.000
*HSP90B1*	1335512822	2	chain A, endoplasmin	0.074	1.000
*HBB*	227343817	15	chain B, crystal structure of dog (Canis Familiaris) hemoglobin	0.087	1.000
*HSP90B1*	159794957	5	chain B, endoplasmin	0.122	1.000
*HSP90B1*	159794959	5	chain B, endoplasmin	0.122	1.000
*HSP90B1*	1258500452	5	chain B, endoplasmin	0.122	1.000
*HSP90B1*	1379069777	2	chain B, endoplasmin	0.074	1.000
*HSP90B1*	159794950	3	chain G, endoplasmin	0.077	1.000
*DSG1*	1418314253	3	desmoglein-1 [*Canis lupus dingo*]	0.069	1.000
*DSG1*	545504019	3	desmoglein-1 isoform X1 [*Canis lupus familiaris*]	0.069	1.000
*DSG1*	4101628	3	desmoglein-1 precursor [*Canis lupus familiaris*]	0.069	1.000
*HSP90B1*	50979166	5	endoplasmin precursor [*Canis lupus familiaris*]	0.122	1.000
*FN1*	1418343247	4	fibronectin isoform X1 [*Canis lupus dingo*]	−0.029	1.000
*FN1*	928182521	4	fibronectin isoform X1 [*Canis lupus familiaris*]	−0.029	1.000
*FN1*	1418343265	4	fibronectin isoform X10 [*Canis lupus dingo*]	−0.029	1.000
*FN1*	928182523	4	fibronectin isoform X10 [*Canis lupus familiaris*]	−0.029	1.000
*FN1*	1418343267	5	fibronectin isoform X11 [*Canis lupus dingo*]	−0.029	1.000
*FN1*	928182519	5	fibronectin isoform X11 [*Canis lupus familiaris*]	−0.029	1.000
*FN1*	1418343249	4	fibronectin isoform X2 [*Canis lupus dingo*]	−0.029	1.000
*FN1*	1239982239	4	fibronectin isoform X2 [*Canis lupus familiaris*]	−0.029	1.000
*FN1*	1418343251	4	fibronectin isoform X3 [*Canis lupus dingo*]	−0.029	1.000
*FN1*	1239982241	4	fibronectin isoform X3 [*Canis lupus familiaris*]	−0.029	1.000
*FN1*	1418343253	5	fibronectin isoform X4 [*Canis lupus dingo*]	−0.029	1.000
*FN1*	928182507	5	fibronectin isoform X4 [*Canis lupus familiaris*]	−0.029	1.000
*FN1*	1418343255	5	fibronectin isoform X5 [*Canis lupus dingo*]	−0.029	1.000
*FN1*	928182509	5	fibronectin isoform X5 [*Canis lupus familiaris*]	−0.029	1.000
*FN1*	1418343257	4	fibronectin isoform X6 [*Canis lupus dingo*]	−0.029	1.000
*FN1*	1239982243	4	fibronectin isoform X6 [*Canis lupus familiaris*]	−0.029	1.000
*FN1*	1418343259	5	fibronectin isoform X7 [*Canis lupus dingo*]	−0.029	1.000
*FN1*	928182511	5	fibronectin isoform X7 [*Canis lupus familiaris*]	−0.029	1.000
*FN1*	1418343261	4	fibronectin isoform X8 [*Canis lupus dingo*]	−0.029	1.000
*FN1*	1239982245	4	fibronectin isoform X8 [*Canis lupus familiaris*]	−0.029	1.000
*FN1*	1418343263	5	fibronectin isoform X9 [*Canis lupus dingo*]	−0.029	1.000
*FN1*	928182513	5	fibronectin isoform X9 [*Canis lupus familiaris*]	−0.029	1.000
*HBD*	103484123	4	globin, partial [*Canis lupus familiaris*]	−0.602	1.000
*GSTM3*	1239908410	3	glutathione S-transferase Mu 3 isoform X1 [*Canis lupus familiaris*]	0.125	1.000
*HSPB1*	696633650	2	heat shock protein 27, partial [*Canis lupus familiaris*]	0.129	1.000
*HSP90B1*	672890024	5	heat shock protein 90 kDa beta member 1, partial [*Canis lupus familiaris*]	0.122	1.000
*HSPB1*	1418307159	3	heat shock protein beta-1 [*Canis lupus dingo*]	0.129	1.000
*HSPB1*	924442944	3	heat shock protein beta-1 [*Canis lupus familiaris*]	0.129	1.000
*HSPB1*	624685	3	heat shock protein [*Canis lupus familiaris*]	0.129	1.000
*HBB*	1418222274	13	hemoglobin subunit beta [*Canis lupus dingo*]	0.102	1.000
*HYAL1*	545533633	9	hyaluronidase-1 isoform X1 [*Canis lupus familiaris*]	−0.459	1.000
*HYAL1*	1418215838	9	hyaluronidase-1 isoform X2 [*Canis lupus dingo*]	−0.459	1.000
*IGHAC*	46392561	2	immunoglobulin alpha heavy chain constant regin variant A, partial [*Canis lupus familiaris*]	−0.732	1.000
*IGJ; JCHAIN*	19715661	2	immunoglobulin J chain, partial [*Canis lupus familiaris*]	0.052	1.000
*KRT10*	1418337563	6	keratin, type I cytoskeletal 10 isoform X1 [*Canis lupus dingo*]	−0.116	1.000
*KRT10*	928144438	6	keratin, type I cytoskeletal 10 isoform X1 [*Canis lupus familiaris*]	−0.116	1.000
*KRT10*	1418337565	6	keratin, type I cytoskeletal 10 isoform X2 [*Canis lupus dingo*]	−0.116	1.000
*KRT10*	928144440	6	keratin, type I cytoskeletal 10 isoform X2 [*Canis lupus familiaris*]	−0.116	1.000
*KRT10*	1418337567	6	keratin, type I cytoskeletal 10 isoform X3 [*Canis lupus dingo*]	−0.116	1.000
*KRT10*	928144442	6	keratin, type I cytoskeletal 10 isoform X3 [*Canis lupus familiaris*]	−0.116	1.000
*LTF*	559767226	35	lactotransferrin precursor [*Canis lupus familiaris*]	0.059	1.000
*LTF*	339305349	2	lactotransferrin, partial [*Canis lupus familiaris*]	0.070	1.000
*LRG1*	1418218016	4	leucine-rich alpha-2-glycoprotein [*Canis lupus dingo*]	−0.066	1.000
*LYZ*	665505916	9	lysozyme C precursor [*Canis lupus familiaris*]	−0.934	1.000
*MYH14*	1418303612	2	myosin-14 isoform X1 [*Canis lupus dingo*]	0.544	1.000
*MYH14*	359318588	2	myosin-14 isoform X1 [*Canis lupus familiaris*]	0.544	1.000
*MYH14*	1418303616	2	myosin-14 isoform X2 [*Canis lupus dingo*]	0.544	1.000
*MYH14*	1239887368	2	myosin-14 isoform X2 [*Canis lupus familiaris*]	0.544	1.000
*MYH14*	1418303618	2	myosin-14 isoform X3 [*Canis lupus dingo*]	0.544	1.000
*MYH14*	1239887370	2	myosin-14 isoform X3 [*Canis lupus familiaris*]	0.544	1.000
*MYH14*	1418303620	2	myosin-14 isoform X4 [*Canis lupus dingo*]	0.544	1.000
*MYH14*	1239887372	2	myosin-14 isoform X4 [*Canis lupus familiaris*]	0.544	1.000
*PEBP1*	114326321	4	phosphatidylethanolamine-binding protein 1 [*Canis lupus familiaris*]	−0.063	1.000
*PEBP4*	1418230432	4	phosphatidylethanolamine-binding protein 4 isoform X1 [*Canis lupus dingo*]	0.265	1.000
*PEBP4*	345790561	3	phosphatidylethanolamine-binding protein 4 isoform X1 [*Canis lupus familiaris*]	0.265	1.000
*PEBP4*	1418230422	3	phosphatidylethanolamine-binding protein 4 isoform X2 [*Canis lupus dingo*]	0.278	1.000
*PEBP4*	1239961995	2	phosphatidylethanolamine-binding protein 4 isoform X2 [*Canis lupus familiaris*]	0.278	1.000
*PEBP4*	1418230424	2	phosphatidylethanolamine-binding protein 4 isoform X3 [*Canis lupus dingo*]	−0.267	1.000
*PEBP4*	1239961997	2	phosphatidylethanolamine-binding protein 4 isoform X3 [*Canis lupus familiaris*]	−0.267	1.000
*PEBP4*	1418230426	4	phosphatidylethanolamine-binding protein 4 isoform X4 [*Canis lupus dingo*]	0.265	1.000
*PEBP4*	1239961999	3	phosphatidylethanolamine-binding protein 4 isoform X4 [*Canis lupus familiaris*]	0.265	1.000
*PEBP4*	1418230428	4	phosphatidylethanolamine-binding protein 4 isoform X5 [*Canis lupus dingo*]	0.265	1.000
*PEBP4*	1239962001	3	phosphatidylethanolamine-binding protein 4 isoform X5 [*Canis lupus familiaris*]	0.265	1.000
*PEBP4*	1418230430	4	phosphatidylethanolamine-binding protein 4 isoform X6 [*Canis lupus dingo*]	0.265	1.000
*PEBP4*	345790559	3	phosphatidylethanolamine-binding protein 4 isoform X6 [*Canis lupus familiaris*]	0.265	1.000
*N/A*	1239884102	4	phospholipase A2 inhibitor and Ly6/PLAUR domain-containing protein, partial [*Canis lupus familiaris*]	−1.061	1.000
*N/A*	1418270515	2	poly(U)-specific endoribonuclease [*Canis lupus dingo*]	0.134	1.000
*N/A*	1239968362	2	poly(U)-specific endoribonuclease [*Canis lupus familiaris*]	0.134	1.000
*N/A*	1239931268	2	proteoglycan 4-like [*Canis lupus familiaris*]	0.054	1.000
*IQGAP1*	1418331169	3	ras GTPase-activating-like protein IQGAP1 [*Canis lupus dingo*]	0.226	1.000
*LYZ*	8928189	9	RecName: Full = Lysozyme C, spleen isozyme; AltName: Full = 1,4-beta-N-acetylmuramidase C	−0.934	1.000
*SCGB1D1*	1418504468	2	secretoglobin family 1D member 2-like [*Canis lupus dingo*]	1.105	1.000
*SCGB1D1*	545531237	2	secretoglobin family 1D member 2-like [*Canis lupus familiaris*]	1.105	1.000
*SH3BGRL3*	345794456	2	SH3 domain-binding glutamic acid-rich-like protein 3 [*Canis lupus familiaris*]	−0.011	1.000
*SCGB1A1*	922664320	2	uteroglobin, partial [*Canis lupus familiaris*]	−0.130	1.000
*N/A*	1239959951	2	vomeromodulin-like [*Canis lupus familiaris*]	0.098	1.000
